# Body reconstruction and size estimation of plesiosaurs

**DOI:** 10.7717/peerj.21146

**Published:** 2026-04-14

**Authors:** Ruizhe Jackevan Zhao

**Affiliations:** Department of Mathematics, Northwest University, Xi’an, China

**Keywords:** Ribcage restoration, Plesiosaurs, Body size estimation, Cross-sectional method, Biological scaling, Skeletal reconstruction, Volumetric-density, Marine reptiles, Regression, Body mass

## Abstract

**Background:**

Plesiosaurs were a clade of Mesozoic aquatic reptiles exhibiting high diversity in neck length. Although their body sizes have long attracted scientific and public attention, mass estimates grounded in rigorous skeletal reconstructions are notably lacking. Existing data often rely on historical museum mounts or outdated illustrations from old literature, casting doubt on their reliability. The body masses of many species also remain unknown to date.

**Methods:**

To bridge this knowledge gap, the present study employs a hybrid paradigm: (1) establish a standardized protocol for accurate skeletal reconstruction of plesiosaurs, with particular emphasis on deriving ribcage morphology from dorsal rib orientation; (2) uniformly apply this protocol to build multiple plesiosaur models spanning multiple clades, whose body masses are estimated using the cross-sectional method, a recently developed volumetric-density approach for mass estimation; and (3) identify reliable skeletal mass proxies by means of regression analysis. Regression models were also employed to estimate the dimensions of unpreserved skeletal elements. Model fit and predictive accuracy were assessed using sample-size corrected Akaike Information Criterion values and per cent prediction errors, respectively.

**Results:**

Rigorous body reconstructions were developed for 27 taxa covering all major plesiosaur clades, with body mass estimates ranging from 79 kg to 12,824 kg. This range sufficiently captures the size diversity seen across most plesiosaurs. These reconstructions were used to evaluate the performance of various skeletal elements as body size proxies. Among the tested metrics, trunk length and the mean volume of dorsal vertebral centrum were identified as the most robust predictors of plesiosaur body mass.

**Discussion:**

Critiques of the hybrid approaches often focus on inconsistent modeling criteria and limited taxonomic sampling. The present framework seeks to mitigate these concerns through uniform reconstruction standards applied across multiple plesiosaur clades. Potential uncertainties in soft-tissue restoration and limitations of the mass equations are explicitly addressed. By providing both rigorous skeletal reconstructions and practical mass-estimation tools, this work narrows the knowledge gap regarding the body masses of Mesozoic aquatic reptiles, and facilitates subsequent biomechanical and macroevolutionary studies on plesiosaurs.

## Introduction

Body size has long been recognized as a fundamental determinant of numerous biological traits (Bonner, 2006; McClain & Boyer, 2009; Campione & Evans, 2020; Orkney & Hedrick, 2024). Understanding size evolution in extinct animals not only reveals the selective pressures and ecological opportunities they encountered over deep time (Brown & Maurer, 1986), but also establishes references for predicting how extant species may respond to environmental change (Shimada et al., 2025). Among various size metrics, body mass is especially important because it is directly linked to many physiological properties such as metabolic rate and locomotor speed (Taylor, Schmidt-Nielsen & Raab, 1970; White & Seymour, 2003; Hirt et al., 2017). However, estimating body mass in extinct animals remains highly contentious. Different studies, often using disparate criteria and methods, can yield markedly divergent mass estimates for the same individual or species (*e.g.*, Gunga et al., 2008; Hurrell, 2019; Motani & Pyenson, 2024; Wright, Cavanaugh & Pierce, 2024). This inconsistency highlights the importance of developing a standardized body mass estimation framework for specific extinct clades, as such standardization enables reliable comparisons of relative body size among species in biomechanical or macroevolutionary analyses (Campione & Evans, 2012; also see the methodologies in Benson et al., 2014, Benson et al., 2018).

One of the primary challenges in estimating body mass for extinct animals is the fragmentary nature of the fossil record. To address this issue, the scaling approaches are often employed to estimate the dimensions of missing anatomical elements or the body masses directly (Hopkins, 2018; Gayford et al., 2024). The underlying assumption of the latter (namely extant scaling (ES) approaches) is that the relationships between certain skeletal measurements and body mass summarized from extant species should also apply to extinct clades (Anderson, Hall-Martin & Russell, 1985; Campione et al., 2014). While the ES approaches provide computational efficiency (Campione & Evans, 2020), the validity of this shared assumption remains unknown. Many previous studies have cautioned that when mass equations are derived exclusively from extant taxa, their reliability for inferring body mass in extinct species is questionable (*e.g.*, Hutchinson, Ng-Thow-Hing & Anderson, 2007; Bates et al., 2015; Romano et al., 2023). Beyond this fundamental concern, other limitations inherent in scaling approaches have also been highlighted in previous studies (*e.g.*, Wright, Cavanaugh & Pierce, 2024; Gayford et al., 2024).

Another category of body mass estimation methods is the volumetric-density (VD) approaches. The VD approaches employ various technical procedures to convert physical, 2D, or 3D models into volume estimates, which are then transformed into body mass by multiplication with assumed body densities (see Campione & Evans, 2020 and references therein). Despite the recent advancement of 3D modeling techniques, their application in paleobiology remains limited in taxonomic coverage due to the substantial time and resources required for accurate skeletal reconstruction, as well as limited access to globally dispersed fossil specimens. In contrast, the lower time investment associated with 2D modeling has promoted the widespread application of planar models and stimulated ongoing methodological advancement (*e.g.*, Motani, 2023). One recently developed 2D approach was the cross-sectional method (CSM; Zhao, 2024). The CSM divides a 2D model into several slabs, and assumes linear transitions in cross-sectional shape and size at a small scale. Unlike methods that rely on assumed cross-sectional geometries (*e.g.*, ellipse, Hurlburt, 1999; Henderson, 1999), the CSM can accommodate any cross-sectional shape and yields more accurate estimates. A critical step in applying the CSM to extinct vertebrates is to determine the transverse ribcage cross-sections from rigorous skeletal reconstructions. These reconstructions themselves form the foundational basis for all VD approaches (Paul, 2019).

Plesiosaurs were a clade of Mesozoic aquatic reptiles with various neck lengths, which were traditionally divided into two morphotypes: the long-necked, small-headed “plesiosauromorph” and the short-necked, large-headed “pliosauromorph” (O’Keefe, 2002). Despite sustained scientific and public interest in plesiosaur body size (*e.g.*, the “25 m *Liopleurodon*” in *Walking with Dinosaurs*), body mass estimations for them remain scarce, most having been derived as secondary results from biomechanical studies (Henderson, 2006; Troelsen et al., 2019; Gutarra et al., 2022; Henderson, 2024). Many of these previous estimates rely heavily on decades-old museum mounts or outdated reconstructions from historical literature. These sources often lack detailed descriptions of the skeletal reconstruction process, making it difficult to evaluate the reliability of the resulting models. To date, the only study focused specifically on size estimation is the unpublished PhD thesis of McHenry (2009), which calculated body lengths for several thalassophonean pliosaurs. However, the accompanying body volumes were derived from a commercial model and did not account for interspecific differences in body proportions. While multiple plesiosaur skeletal reconstructions were presented in one popular science publication (Paul, 2022), the corresponding body mass estimation procedure was incompletely described.

Although only a few body mass estimates for plesiosaurs are based on rigorous skeletal reconstructions, cumulative contributions from previous research have established a solid foundation for such attempts. Some plesiosaurs possessed the highest number of cervical vertebrae among all vertebrates (Kubo, Mitchell & Henderson, 2012; Sachs, Kear & Everhart, 2013), a feature that has attracted considerable research interest. Comprehensive vertebral measurements of multiple plesiosaur specimens are available in the literature (*e.g.*, Welles, 1943; Welles, 1952; McHenry, 2009; Sachs, Hornung & Kear, 2016; Sachs & Madzia, 2025), and in turn enable reliable body length estimation. The body length estimation framework proposed for pliosaurs by McHenry (2009) is also applicable to all plesiosaurs. Furthermore, the preservation of soft tissue impressions in several plesiosaur fossils provides extra insight into their body shape *in vivo* (Vincent et al., 2017; Frey et al., 2017; Sachs et al., 2025; Marx et al., 2025).

Some studies adopted a hybrid approach to estimate body masses of extinct vertebrates, by developing regression formulae based on either VD estimates or composite datasets that include both extinct and extant taxa (*e.g.*, Seebacher, 2001; Therrien & Henderson, 2007). The hybrid approaches can mitigate the limitations of both the VD and ES approaches: (1) Applying these regression formulae avoids the considerable time investment required by the VD approaches; (2) Because the backbone datasets are calibrated using VD estimates of specific extinct taxa, the resulting formulae are (at least partially) ensured to be applicable to these extinct clades (see Campione & Evans, 2020 for a review of the advantages and disadvantages). Therefore, the hybrid approach holds potential for rapid and reliable comparison of body mass in extinct clades. A principal critique of the hybrid approach highlights inconsistent reconstruction standards in the underlying VD datasets, coupled with inadequate taxonomic sampling (Campione et al., 2014; Campione & Evans, 2020). Ideally, these shortcomings could be mitigated by proposing and implementing a uniform set of reconstruction criteria across multiple representative taxa within a specific clade of extinct vertebrates. This study aims to establish a hybrid methodological framework for body mass estimation in plesiosaurs by (1) proposing a standardized skeletal modeling protocol, (2) applying it across a phylogenetically broad range of plesiosaur taxa, and subsequently (3) using regression models based on the resulting body mass estimates to identify anatomical proxies that reliably predict plesiosaur body mass. By identifying these effective body mass proxies, the body mass equations provided in this study would enable future researchers to rapidly estimate body mass across a wide range of plesiosaur taxa, thereby facilitating subsequent biomechanical and body size evolution studies.

## Materials and Methods

### Body reconstruction of plesiosaurs

As outlined in the Introduction, the hybrid approach depends on precise modeling of multiple species within a consistent framework, and requires the taxonomic sample to be broadly representative of the entire clade. To address this requirement, this study proposes a standardized protocol for plesiosaur reconstruction, highlighting the role of ribcage restoration in estimating the cross-sectional contours. The general workflow is summarized below. Further specifics such as additional criteria, software procedures, and three worked examples are available in Supplementary Material, allowing the methodology presented here to be reproduced.

**Figure 1:**
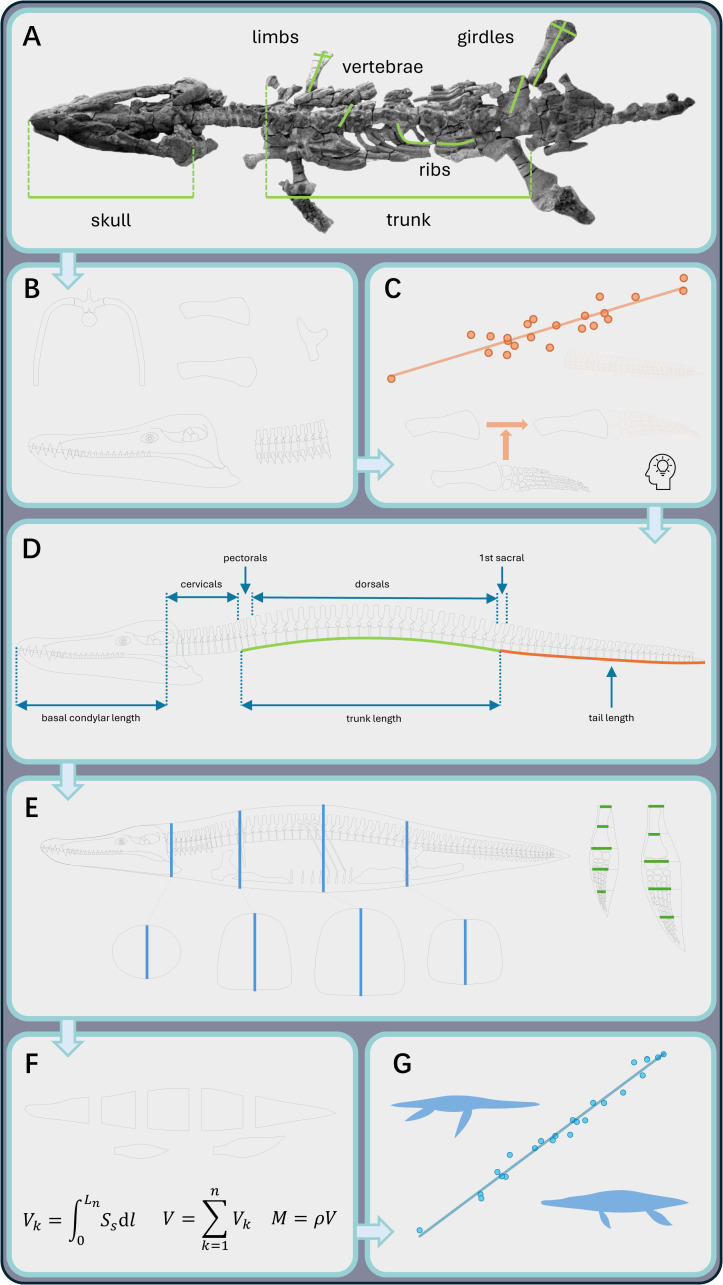
The workflow for establishing a hybrid framework to estimate body mass in plesiosaurs. (A) Acquire data from fossil specimens (illustrated here using the modified image of *Sachicasaurus vitae*, Paramo-Fonseca, Benavides-Cabra & Gutierrez, 2018, CC BY 4.0). (B) Create lateral-view silhouettes of skeletal elements. (C) Estimate the dimensions of missing fossil elements through regression formulae and/or comparisons with closely related taxa. (D) Reconstruct the main body axis, including the dimensions and shape of the skull and vertebral column. (E) Reconstruct the ribcage in both lateral view and transverse cross-sections jointly, then restore the soft-tissue outlines of the main body axis and the flippers. (F) Estimate the body mass using the cross-sectional method (Zhao, 2024). (G) Establish body mass equations from the mass estimates of multiple species. Elements in this figure are not to scale.

Figure 1A illustrates the schematic data acquisition procedure, using the holotype of *Sachicasaurus vitae* as an example (Paramo-Fonseca, Benavides-Cabra & Gutierrez, 2018). At this stage, the measurements essential for reconstruction were collected, including skull size, trunk length, vertebral dimensions, rib arc length (RAL), sizes of the pectoral and pelvic girdle elements, and limb dimensions (see Fig. S1 for measurement criteria). Whenever possible, this study relied on published data from the original description. For dimensions not previously reported, values were obtained through a photogrammetric method in AutoCAD (Shoukry & Pandey, 2020), based on strictly oriented photographs obtained from colleagues or the literature. All planar models were created in AutoCAD, with the unit precision set to 0.0001 mm. The skeletal elements were first drafted as individual structures (Fig. 1B). For unpreserved anatomical structures, their dimensions were restored based on regression formulae or body proportions of closely related taxa (Fig. 1C; see below). These individual components were then assembled into a composite model.

The main body axis was first assembled, incorporating all anatomical elements that contribute to total body length, including the skull, vertebrae, and intervertebral cartilage (Fig. 1D). Intervertebral cartilage is recognized as a significant contributor to body length in plesiosaurs (McHenry, 2009), and its dimensions can be inferred from articulated fossils. Troelsen (2018) suggested that intervertebral distance increases in proportion with vertebral dimensions, making proportional values a more reliable quantitative standard than absolute measurements. Moreover, the dimensions of intervertebral cartilage may vary among cervical vertebrae at different positions within an individual (Roberts et al., 2020). Therefore, this study infers the average relative dimension of intercervical cartilage based on all cervical vertebrae, or as many as possible when the series is incomplete or partially disarticulated. The dimensions of intercervical cartilage also vary across different plesiosaur clades. For example, the increased intercervical distance in short-necked taxa may compensate for the reduction in cervical count, as proposed by Wintrich et al. (2019). Quantitative criteria for intervertebral spacing in each plesiosaur clade are provided in Supplementary Material.

**Figure 2:**
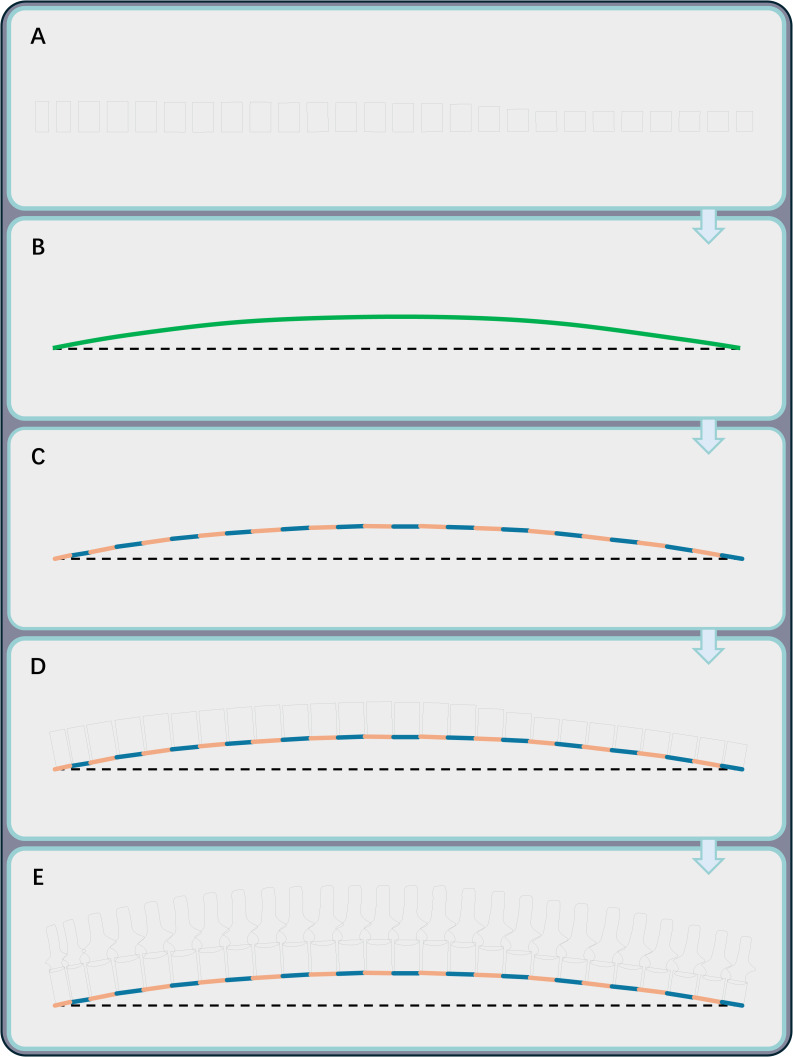
The workflow for restoring the spinal curvature of the trunk region. (A) Represent each vertebral centrum by a rectangle with side lengths corresponding to its centrum length and height. Other vertebral centra (*e.g.*, cervicals) can be reconstructed in the same way. (B) Draw a dashed horizontal line segment representing the trunk length, with a green curve representing the spinal curvature connecting its endpoints. (C) Use a polyline to approximate the curve. (D) Align the vertebrae to the polyline. (E) Add additional structures such as the neural spines to the vertebral centra.

Spinal curvature is a pivotal indicator of body shape and requires reconstruction for fossil specimens with disarticulated vertebral columns. The articulated cervical series of each plesiosaur model was restored as a gentle sigmoidal curve, following the morphology described by Carpenter et al. (2010). For the trunk region, a mathematical model based on vertebral wedging-angles, originally developed for primates, was applied to several plesiosaur taxa in an unpublished thesis (Richards, 2011). However, this method demands a complete, disarticulated vertebral series unaffected by taphonomic distortion, in addition to multiple measurements of the vertebral centra. Given these practical constraints, this study used an alternative approach. Like most other amniotes, plesiosaurs exhibit a regionalized vertebral column (Andrews, 1910). In some well-preserved fossil specimens, the girdle elements align with these regional boundaries: the anterior margin of scapula corresponds to the first pectoral vertebra, and the acetabulum aligns with the second sacral vertebra (*e.g.*, *Plesionectes longicollum* SMNS 51945, Sachs & Madzia, 2025: fig. 1; *“Monquirasaurus” boyacensis* MJACM 1, Noè & Gómez-Pérez, 2022: fig. 8; *Albertonectes vanderveldei* TMP 2007.011.0001, Kubo, Mitchell & Henderson, 2012: fig. 2). Using this anatomical correlation, the trunk length, defined as the distance from the anterior margin of the scapula to the acetabulum, was first drawn as a horizontal segment during modeling (Fig. 2B). A curve was then created to connect the endpoints of this segment (Fig. 2B) and iteratively adjusted in AutoCAD until its length matched the combined lengths of the relevant vertebral elements (*i.e.,* the pectoral and dorsal vertebrae, the first sacral vertebra, and intervertebral cartilage; Fig. 1D). This curve was approximated by a polyline, with each segment representing the combined length of a corresponding vertebra and the intervertebral cartilage (Fig. 2C). The reconstructed vertebral centra (approximated using rectangles, Fig. 2A) were subsequently aligned along this curve (Fig. 2D). Other structures such as the neural spines were then added in the reconstruction (Fig. 2E). Detailed software commands for reconstructing spinal curvature are provided in Supplementary Material. Similar to the neck region, the tail was reconstructed as a gently curved structure to facilitate subsequent volumetric calculation (Fig. 1D).

**Figure 3:**
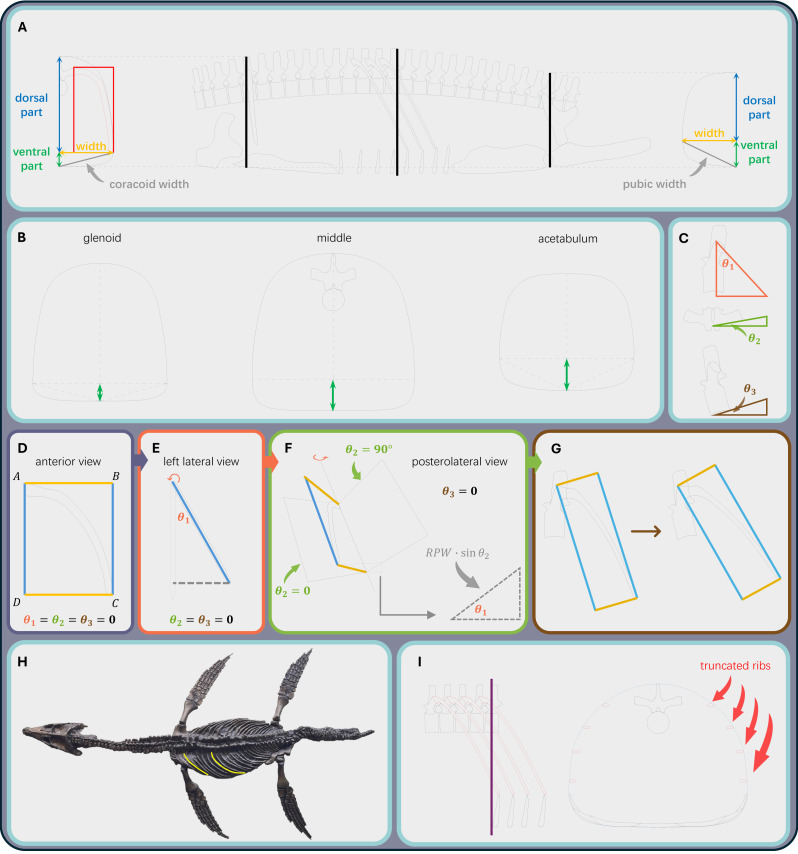
The workflow for restoring the ribcage and body cross-sections. (A) The reconstructed ribcage in lateral view showing the positions of the three ribcage cross-sections, and the glenoid and acetabulum cross-sections in front view. The rectangle represents the rib plane after rotation. (B) The outlines of the three ribcage cross-sections in front view. The double-headed arrows represent the heights of the ventral parts. (C) The method to infer angles *θ*_1_, *θ*_2_, and *θ*_3_ from vertebral morphology. (D) The vertically orientated rib plane (*θ*_1_ = *θ*_2_ = *θ*_3_ = 0) showing the rib plane height (RPH) and rib plane width (RPW). (E) Lateral view of the rib plane that rotates around the AB axis by *θ*_1_ degrees. (F) Posterolateral view of the rib plane that rotates around the AD axis by *θ*_2_ degrees. (G) Lateral view of the costovertebral system that rotates around the mediolateral axis by *θ*_3_ degrees. (H) The photo of *Rhomaleosaurus cramptoni* NHMUK PV R34 (cast of holotype. Source credit: Hongyi (Frederick) Jiang). The highlighted curves represent the arc lengths of the rib dorsal to the glenoid cavity and the longest rib, respectively. (I) Schematic images showing the method to determine the dorsal outline of the middle cross-section from truncated ribs (see Supplementary Material for step-by-step software operations).

The cross-sectional method (CSM) relies on transverse cross-sectional profiles to calculate body volume (Zhao, 2024). An ellipse was employed to approximate the cross-sectional profile of the skull at the quadrate level (Fig. 1E). The major and minor axes of the ellipse were defined as the width and height of the skull at that position (estimated from a close relative if the skull is not preserved, see Table S1 for details). Three cross-sections were established to define the ribcage outline of a plesiosaur, assuming a gradual transition in body shape between them (Figs. 1E; 3A and 3B): (1) the glenoid cross-section, representing the vertical plane intersecting the glenoid cavity of the pectoral girdle; (2) the acetabulum cross-section, representing the vertical plane intersecting the acetabulum of the pelvic girdle; and (3) a middle cross-section, positioned midway between the other two. Each of the three cross-sections was divided into dorsal and ventral parts (Figs. 3A and 3B). The dorsal parts of the cross-sections contain the vertebrae and dorsal ribs, whereas the ventral parts contain the coracoids, pubes, and gastralia, respectively. A key step in reconstructing the cross-sectional profiles involves restoring the spatial orientation of the dorsal ribs, which was achieved through a mathematical procedure presented below.

All known plesiosaurs possess single headed dorsal ribs that articulate exclusively with the transverse processes (Andrews, 1910). This consistent feature allowed O’Keefe et al. (2011) and Richards (2011) to infer differences in body shape among several cryptoclidids based on variations in their transverse process morphology. Different from their schemes, this study incorporates three distinct rib slant angles and infers rib orientation through composite 3D rotations (Fig. 3C). Angle *θ*_1_ ∈ [ − 90^∘^, 90^∘^] quantifies the deviation of pump-handle rotation (*i.e.,* around a mediolateral axis) from the vertical plane, while angle *θ*_2_ ∈ [0^∘^, 90^∘^] quantifies the deviation of bucket-handle rotation (*i.e.,* around a dorsoventral axis) from the vertical plane. Both *θ*_1_ and *θ*_2_ were inferred from the morphology of the transverse processes (Fig. 3C). Angle *θ*_3_ ∈ [ − 90^∘^, 90^∘^] quantifies the deviation of vertebral centrum from the horizontal plane (Fig. 3C). The magnitudes of angles *θ*_1_ and *θ*_2_ differ among plesiosaur species (*e.g.*, max angle *θ*_2_ = 9° in *Cryptoclidus eurymerus*, Richards, 2011; and 30° in *Hydrotherosaurus alexandrae*, Welles, 1943). The gradual change in rib slant angles along the vertebral column, a pattern documented in well-preserved fossils and noticed by prior studies, appears common among plesiosaurs (*e.g.*, Andrews, 1910; O’Keefe et al., 2011; Sachs et al., 2025). Both angles *θ*_1_ and *θ*_2_ increase gradually from 0° in anterior dorsals to their maximum values in the middle to posterior part of the dorsal region. Likewise, rib length typically increases gradually across the anterior half of the dorsal series, then decreases in the posterior dorsal region (Williston, 1903; Welles, 1943).

The general mathematical model for rib rotation is first described, and the detailed procedures for reconstructing cross-sectional outlines from rib orientation are presented in the following sections. The dorsal rib is first simplified as a 2D structure, and assumed to lie within a vertical plane (Fig. 3D). The minimum rectangle containing the rib is drawn and reveals its height and width(termed here as the rib plane height (RPH) and the rib plane width (RPW)). The four vertices of the rectangle are labeled A, B, C, and D sequentially. Angle *θ*_1_ is considered first, and the rib plane is rotated around the AB axis by *θ*_1_ degrees (Fig. 3E). Then the RPH and RPW projected to the vertical plane in this moment (RPH_*P*_1__ and RPW_*P*_1__, respectively) are

(1) \begin{eqnarray*}{\mathrm{RPH}}_{{P}_{1}}=\mathrm{RPH}\cdot \cos \nolimits {\theta }_{1} {\mathrm{RPW}}_{{P}_{1}}=\mathrm{RPW}.\end{eqnarray*}

Then the rib plane is flipped around the AD axis by *θ*_2_ degrees (Fig. 3F), and the projected height (RPH_*P*_2__) and width (RPW_*P*_2__) are given by

(2) \begin{eqnarray*}{\mathrm{RPH}}_{{P}_{2}}={\mathrm{RPH}}_{{P}_{1}}-\mathrm{RPW}\cdot \sin \nolimits {\theta }_{2}\cdot \sin \nolimits {\theta }_{1} {\mathrm{RPW}}_{{P}_{2}}=\mathrm{RPW}\cdot \cos \nolimits {\theta }_{2}.\end{eqnarray*}

After the determination of rib orientation inferred from the vertebral morphology, the costovertebral system is rotated around the mediolateral axis by *θ*_3_ degrees (Fig. 3G). Then the projected height and width are

(3) \begin{eqnarray*}{\mathrm{RPH}}_{{P}_{3}}={\mathrm{RPH}}_{{P}_{2}}\cdot \cos \nolimits {\theta }_{3} {\mathrm{RPW}}_{{P}_{3}}={\mathrm{RPW}}_{{P}_{2}}.\end{eqnarray*}

Among the three cross-sections of the ribcage, the glenoid cross-section was first established (Fig. 3A). Where preservation allows, the arc length of the ribs dorsal to the glenoid can be measured *via* the photogrammetric method. However, the ribs in many plesiosaur fossils are often poorly preserved or unmeasurable due to the mounted state (*e.g.*, *Pliosaurus funkei*, Knutsen, Druckenmiller & Hurum, 2012; *Vectocleidus pastorum*, Benson et al., 2013; *Thalassomedon haningtoni*, Sachs et al., 2021). To address the general scarcity of complete rib sequences, this study introduces a “rib coefficient”, defined as the ratio of the arc length of the rib dorsal to the glenoid to the maximum rib arc length observed in the specimen (see Fig. 3H). For each plesiosaur clade, one representative specimen with a relatively complete rib sequence was selected to serve as a reference for closely related species (see Supplementary Material for quantitative criteria). As noted above, angles *θ*_1_ and *θ*_2_ both increase from 0^∘^ along the dorsal series. Since the glenoid cross-section often aligns with the most anterior dorsal vertebrae, only angle *θ*_3_ was considered for this section. The corresponding rib plane was first reoriented in AutoCAD, then its frontal projection was aligned with the planar profile of the vertebral transverse process (Fig. 3A). A contour curve was subsequently traced along the outer margin of the rib silhouette in this view. Some extra space was incorporated to accommodate the musculature responsible for forelimb elevation (*i.e.*, *m. latissimus dorsi*, Krahl & Witzel, 2021 also see Discussion), resulting in a rounded body outline. The rib and vertebra jointly define both the height and width of the dorsal part of the glenoid cross-section. The coracoid width and half of the cross-sectional width then form the two sides of a right triangle, allowing the height of the ventral part to be calculated *via* the Pythagorean theorem (Fig. 3A). The external contour of the ventral part was derived by adapting the reconstructions of preserved plesiosaur gastralia (*e.g.*, *Cryptoclidus eurymerus*, O’Keefe et al., 2011; *Fluvionectes sloanae*, Campbell et al.. 2021), under the assumption that soft tissues in the ventrolateral pectoral region would have generated a morphologically comparable outline. Notably, the current modeling approach does not incorporate caliper rotation (*i.e.,* around an anteroposterior axis) of the rib, as this parameter is frequently indeterminable from available fossil materials. Nevertheless, any reduction in body width is always compensated by an increase in dorsoventral depth, and the width of the coracoid provides an upper-bound estimate for the body width at the glenoid level.

In the reconstruction protocol adopted in this study, the ventral margins of both the glenoid and acetabulum cross-sections were assumed to lie within a common horizontal line, with the two sections sharing an identical width (Fig. 3A). Therefore, the height of the ventral part of the acetabulum cross-section can also be calculated using the Pythagorean theorem. The total height was determined based on the ribcage reconstruction in lateral view (Fig. 3A). Considering the tendency of posterior dorsal ribs in plesiosaurs to become shorter and distally pointed (Williston, 1903), the contour of the dorsal part of the acetabulum cross-section was generated by modifying the profile of the glenoid cross-section. This adjustment was carried out using the scaling and stretching functions in AutoCAD (see Supplementary Material for step-by-step software operations).

The middle cross-section intersects multiple dorsal ribs (Figs. 1E; 3A and 3I). The ribs transected by this plane were assumed to lie at a uniform dorsoventral level and were assigned a standardized size equal to the maximum rib arc length. In each plesiosaur model, the vertebral column exhibits a gentle dorsal arch (Figs. 1D and 2D); consequently, the *θ*_3_ angles of the middle dorsal vertebrae approximate 0^∘^, which was an assumption incorporated into the reconstruction. The position of the intersection within each truncated rib plane was determined through a sequence of measurement and alignment steps (see Supplementary Material for step-by-step software operations). After that, the intersected width of each rib plane (RPW_*i*_, *i* = 1, 2, …) was measured. The projected width (RPW_*iP*_) was calculated as RPW_*iP*_ = RPW_*i*_⋅cos*θ*_2_ (Eq. (2); also see Fig. S4). After establishing the positions of all intersected ribs, a smooth curve was drawn through these points to reconstruct the outline of the dorsal part (Fig. 3I). The height of the ventral part (Fig. 3B) was calculated using the following equation:

(4) \begin{eqnarray*}\text{middle ventral height}= \frac{ \frac{1}{2} \left( \text{glenoid ventral height}+\text{acetabulum ventral height} \right) }{\text{rib coefficient}} .\end{eqnarray*}

It is important to note that 3D rotations are non-commutative. This means that applying angle *θ*_1_ before *θ*_2_, or *vice versa*, yields different final positions for the ribs and introduces uncertainty into ribcage reconstruction. Nevertheless, this discrepancy is likely negligible, since any reduction in ribcage height would be compensated by a corresponding increase in width, and *vice versa*.

In the 2D reconstructions followed by the cross-sectional method, all limbs are constructed as separate, discrete objects to facilitate volumetric calculations (Fig. 1F). For the plesiosaur models in this study, every limb element was drawn and assembled in AutoCAD (Fig. 1B). Where distal elements were absent, total limb length was estimated from the distal width of the propodial using the equation provided in Sydes (2022 also see below), with skeletal proportions restored based on a closely related species (Fig. 1C). In cases where an entire pair of forelimbs or hindlimbs was missing, flipper lengths were estimated by comparison with congeneric or other closely related species (see Table S1 for details).

Soft tissue reconstruction represents the final modeling step (Fig. 1E). Minimal craniofacial soft tissue was added to the plesiosaur models, while the three ribcage cross-sections were uniformly enlarged by 25%. An additional 5% tail length, accounting for soft tissue, was appended to the posterior end of each model, based on soft tissue traces observed in *Seeleyosaurus guilelmiimperatoris* MB.R.1992 (Sachs et al., 2025). Limb outlines were restored following the protocol of Muscutt (2017): limb length was divided into five equal segments, and relative thickness of soft tissue at the leading and trailing edges of each segment was reconstructed based on penguin morphology (Fig. 1E; see Muscutt, 2017 for illustrated workflow). The rationale for soft tissue restoration criteria is provided in the Discussion.

### Regression analyses

The fragmentary nature of the fossil record frequently complicates the reconstruction of extinct vertebrates, necessitating the estimation of the sizes of missing elements through comparisons with close relatives (*e.g.*, McHenry, 2009; Paul, 2022) or the use of regression equations (Gayford et al., 2024 and references therein). The plesiosaur fossil record exhibits several common patterns of incompleteness: (1) The modeling protocol proposed in this study depends on a highly complete presacral vertebral sequence, yet the skull is not always preserved; (2) Distal caudal vertebrae are frequently absent (*e.g.*, the total caudal count and tail length remain unknown for most thalassophonean pliosaurs; McHenry, 2009; Paramo-Fonseca, Benavides-Cabra & Gutierrez, 2018; Noè & Gómez-Pérez, 2022); and (3) Given the hyperphalangy characteristic of plesiosaur limbs (Krahl, 2021), distal elements are often incomplete, rendering fully preserved limbs exceptionally rare. To address these recurrent issues, this study established regression equations to estimate the skull length (SKL; from neck length and cervical count) and tail length (from trunk length, femur length and distal width) by integrating specimen data across multiple plesiosaur clades.

For limb length estimation, this study adopted a pre-existing regression formula (Sydes, 2022) relating propodial distal width to total limb length. This equation has wide applicability because propodials are among the most commonly preserved elements in plesiosaur fossils. Additionally, another regression formula was developed to predict maximum rib arc length from trunk length, given the critical role of rib dimensions in modeling and the practical difficulty of obtaining rib measurements, as mentioned above.

Regression models were also used to evaluate the performance of various skeletal elements as predictors of body mass (Fig. 1G; see below for body mass calculation), including skull length (SKL)×cervical number (CN), trunk length, humerus length and distal width, femur length and distal width, coracoid length and width, pubic length and maximum width, ischium length and width (see Fig. S1 for measurement criteria). Combined dimensions of dorsal vertebral centra (specifically, mean width × mean height, and mean length × mean width × mean height) served as proxies for the cross-sectional area and volume of vertebral centrum, respectively. Their effectiveness as body mass predictors was also assessed using regression models. The independent variables selected for both reconstruction and body mass estimation were not chosen by random data mining. Instead, their selection was based on a structured rationale informed by prior empirical knowledge, potential functional modules, and available fossil measurement data (see Discussion for details).

In case of Type I errors caused by shared evolutionary history, a plesiosaur phylogeny was incorporated into the regression analyses to correct for autocorrelation. Without accounting for phylogenetic relatedness, the non-independence of species data can create a misleading impression that two variables are significantly associated, even when no true relationship exists (Felsenstein, 1985; Grafen, 1989). A character matrix was selected from Sachs & Madzia (2025), and their maximum parsimony protocol (using implied weighting, *K* = 12) was replicated in TNT 1.6 (Goloboff & Morales, 2023). The strict consensus tree was then summarized and time-calibrated in R 4.3.3 (R Core Team, 2024). Most temporal ranges for the operational taxonomic units (OTUs) were obtained from Madzia & Cau (2020), while those for *Colymbosaurus svalbardensis* and *Ophthalmothule cryostea* were sourced from Roberts et al. (2020). Additional temporal data were retrieved from the Paleobiology Database (https://paleobiodb.org/). Time-calibration was accomplished using the timePaleoPhy() function from the paleotree package version 3.4.7 (Bapst, 2012) under the minimum branch length method. The minimum branch length was set to 1 million years, and the polytomies were randomly resolved during the process.

To analyze size correlations, Phylogenetic Generalized Least Squares (PGLS) and Ordinary Least Squares (OLS) were applied to the same datasets. In addition to the linear models, the skull-neck dataset was also fitted using a nonlinear regression based on a log–logistic function (see Discussion for rationale). Three samples (*Serpentisuchops pfisterae*, *Styxosaurus* SDSM 451, and *Thaumatodracon wiedenrothi*) are absent from the current phylogeny. As they represent only a small fraction of the total sample (3 out of 40), they were excluded from the skull-neck dataset during the first round of model fitting. Subsequently, OLS and the nonlinear model were also applied to the original dataset. Given that *Styxosaurus* SDSM 451 also serves as the basis for one plesiosaur model in this study, PGLS models were compared with OLS models using body mass datasets pruned to exclude this specimen. All measurements were obtained either from published literature or through photogrammetry in AutoCAD, based on strictly oriented photographs from publications or colleagues. The selected variables were first log_10_-transformed prior to the model fitting. The OLS models were fitted using the lm() function implemented in base R stats package (R Core Team, 2024), and the PGLS models were fitted with the gls() function from the nlme package version 3.1-164 (Pinheiro, Bates & R Core Team, 2023). The corBrownian autocorrelation structure was employed to accommodate phylogenetic non-independence. The nonlinear model for skull-neck dataset was fitted by using the drm() function implemented in the drc package version 3.0-1 (Ritz et al., 2015), with a four-parameter log–logistic function. Model performance was compared using sample-size corrected Akaike Information Criterion (AICc) values and mean per cent prediction errors ($\overline{ \left\vert \%\mathrm{PE} \right\vert }$, Smith, 1980; Campione & Evans, 2020). The AICc value quantifies model fit to data, whereas the mean prediction error assesses predictive performance. To compute the $\overline{ \left\vert \%\mathrm{PE} \right\vert }$ value, each specimen was iteratively removed from the dataset as the testing set, with the remaining specimens serving as the training set for regression modeling. The resulting model was then used to predict the value of the testing set (*i.e.,* the removed sample), and the antilog-scaled prediction value was compared with the observed value:

(5) \begin{eqnarray*} \left\vert \%\mathrm{PE} \right\vert = \left\vert \frac{\text{observed value}-\text{predicted value}}{\text{predicted value}} \right\vert \times 100 \overline{ \left\vert \%\mathrm{PE} \right\vert }= \frac{\sum _{i=1}^{N}{ \left\vert \%\mathrm{PE} \right\vert }_{i}}{N} .\end{eqnarray*}

Different from the confidence intervals, the $\overline{ \left\vert \%\mathrm{PE} \right\vert }$ value provides a symmetrical error range when the predicted value is back-transformed to its antilog scale (Campione & Evans, 2020):

(6) \begin{eqnarray*}{\text{predicted value}}_{\text{without log-transformation}}\times \left( 1\pm \frac{\overline{ \left\vert \%\mathrm{PE} \right\vert }}{100} \right) .\end{eqnarray*}

To further evaluate the performance of the variables for body mass prediction, this study also calculated the standard deviation of the $ \left\vert \%\mathrm{PE} \right\vert $ values. A larger standard deviation indicates that the regression model produces substantially different levels of error across species, yielding relatively large errors for some and smaller errors for others. In contrast, a smaller standard deviation suggests that the model performs more consistently across different taxa. The standard deviation is defined as

(7) \begin{eqnarray*}\mathrm{sd} \left( \left\vert \%\mathrm{PE} \right\vert \right) =\sqrt{ \frac{1}{N-1} \sum _{i=1}^{N}{ \left( { \left\vert \%\mathrm{PE} \right\vert }_{i}-\overline{ \left\vert \%\mathrm{PE} \right\vert } \right) }^{2}}.\end{eqnarray*}

### Volumetric computation and mass estimation

**Figure 4:**
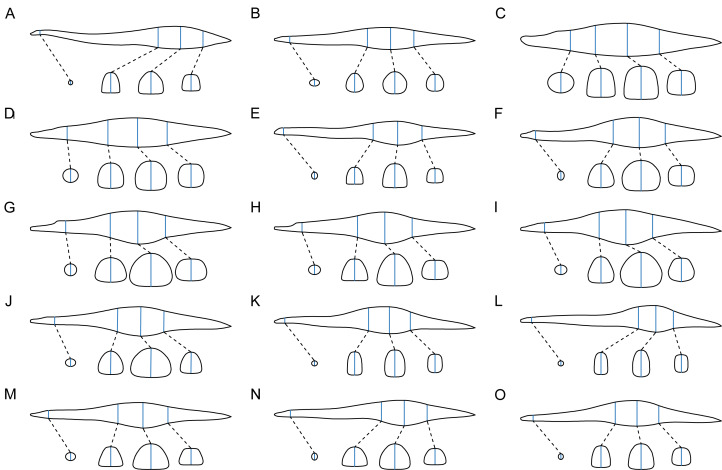
Silhouettes of representative plesiosaur models from different clades, showing the reconstructed body outlines and transverse cross-sections. All models are scaled to the same length. (A) *Thalassomedon haningtoni*. (B) *Aristonectes quiriquinensis*. (C) *Sachicasaurus vitae*. (D) *Liopleurodon ferox* (E) *Abyssosaurus nataliae*. (F) *Cryptoclidus eurymerus*. (G) *Martinectes bonneri*. (H) *Polycotylus latipinnis*. (I) *Meyerasaurus victor*. (J) *Macroplata tenuiceps*. (K) *Seeleyosaurus guilelmiimperatoris*. (L) *Microcleidus tournemirensis*. (M) *Nichollssaura borealis*. (N) *Brancasaurus brancai*. (O) *Plesiopterys wildi*.

Figure 4 shows the representative silhouettes employed to estimate axial body volume across different plesiosaur clades, together with a comparative visualization of morphological divergence in the reconstructed body cross-sections. Collectively, neck elongation appears to correlate with a more slender and dorsoventrally deepened trunk morphology. Volumetric calculation was performed using the cross-sectional method (CSM; Zhao, 2024). The main body of each plesiosaur was partitioned into five slabs by the four transverse cross-sections (Figs. 1E and 1F). A constant shape was assumed for the slab at each end of the body (*i.e.,* the first one and the last one), while others were assumed to possess gradually changing profiles. Each limb was treated as a single slab, and the cross-section of the hydrofoil used in Muscutt (2017) was employed here. Each slab was sliced into 100 subslabs, which far exceeds the number required by stable estimation of the CSM (Zhao, 2024). The model slicing was conducted in AutoCAD, and the final computation was carried out in Excel.

The body mass of each plesiosaur model was estimated by multiplying the calculated volume by an assumed density. Although the body density of aquatic tetrapods can fluctuate with daily behaviors, these animals are presumed to approach neutral buoyancy during specific activities (*e.g.*, during foraging or horizontal transportation, Sato et al., 2013; Gordine, Fedak & Boehme, 2015). Accordingly, the average body density of all plesiosaur models was set to 1.027 g/cm^3^, matching the density of surface seawater. The same criterion has been adopted in previous studies focusing on other marine tetrapods (*e.g.*, Motani & Pyenson, 2024).

## Results

### Regression equations for missing elements

All regression models for missing elements encompass phylogenetically diverse samples, including representatives from all major plesiosaur clades, suggesting broad applicability across Plesiosauria. For the trunk-rib (*N* = 24) dataset, the Phylogenetic Generalized Least Squares (PGLS) model reveals a significant correlation between maximum rib arc length (max RAL) and trunk length (*P* < 0.001), and the Ordinary Least Squares model (OLS; *R*^2^ = 0.9030) demonstrates better fit and predictive performance, as indicated by lower AICc and $\overline{ \left\vert \%\mathrm{PE} \right\vert }$ values (a pattern consistent with some previous studies; Benson et al., 2018; Campione & Evans, 2020).

(8) \begin{eqnarray*}\mathrm{PGLS:}{\log \nolimits }_{10} \left( \text{max RAL} \right) =1.0019{\log \nolimits }_{10} \left( \mathrm{trunk} \right) -0.4365 \mathrm{AICc}=-36.85 \overline{ \left\vert \%\mathrm{PE} \right\vert }=15.22\end{eqnarray*}

(9) \begin{eqnarray*}\mathrm{OLS:}{\log \nolimits }_{10} \left( \text{max RAL} \right) =1.0123{\log \nolimits }_{10} \left( \mathrm{trunk} \right) -0.5068 \mathrm{AICc}=-54.88 \overline{ \left\vert \%\mathrm{PE} \right\vert }=14.29.\end{eqnarray*}

Similarly, PGLS models reveal a significant correlation (*P* < 0.001) between tail length and each of the following: femur length (FemL), femur distal width (FemW), and trunk length. All corresponding OLS models were fitted using the same set of species (*N* = 22). Consequently, their *R*^2^ (0.6098 in FemL-tail regression; 0.7217 in FemW-tail; 0.8175 in trunk-tail) and $\overline{ \left\vert \%\mathrm{PE} \right\vert }$ values can be directly compared, reflecting the relative predictive power of each variable for estimating tail length.

(10) \begin{eqnarray*}\mathrm{PGLS:}{\log \nolimits }_{10} \left( \mathrm{tail} \right) =0.9706{\log \nolimits }_{10} \left( \mathrm{FemL} \right) +0.6887 \mathrm{AICc}=-26.76 \overline{ \left\vert \%\mathrm{PE} \right\vert }=19.80\end{eqnarray*}

(11) \begin{eqnarray*}\mathrm{PGLS:}{\log \nolimits }_{10} \left( \mathrm{tail} \right) =0.7780{\log \nolimits }_{10} \left( \mathrm{FemW} \right) +1.4225 \mathrm{AICc}=-28.54 \overline{ \left\vert \%\mathrm{PE} \right\vert }=16.32\end{eqnarray*}

(12) \begin{eqnarray*}\mathrm{PGLS:}{\log \nolimits }_{10} \left( \mathrm{tail} \right) =0.8434{\log \nolimits }_{10} \left( \mathrm{trunk} \right) +0.4895 \mathrm{AICc}=-41.25 \overline{ \left\vert \%\mathrm{PE} \right\vert }=12.77\end{eqnarray*}

(13) \begin{eqnarray*}\mathrm{OLS:}{\log \nolimits }_{10} \left( \mathrm{tail} \right) =0.8074{\log \nolimits }_{10} \left( \mathrm{FemL} \right) +1.0675 \mathrm{AICc}=-34.94 \overline{ \left\vert \%\mathrm{PE} \right\vert }=19.56\end{eqnarray*}

(14) \begin{eqnarray*}\mathrm{OLS:}{\log \nolimits }_{10} \left( \mathrm{tail} \right) =0.8231{\log \nolimits }_{10} \left( \mathrm{FemW} \right) +1.2899 \mathrm{AICc}=-42.38 \overline{ \left\vert \%\mathrm{PE} \right\vert }=17.42\end{eqnarray*}

(15) \begin{eqnarray*}\mathrm{OLS:}{\log \nolimits }_{10} \left( \mathrm{tail} \right) =0.8414{\log \nolimits }_{10} \left( \mathrm{trunk} \right) +0.4803 \mathrm{AICc}=-51.66 \overline{ \left\vert \%\mathrm{PE} \right\vert }=13.43.\end{eqnarray*}

Each OLS model produces a significantly lower AICc value than its corresponding PGLS counterpart, though the OLS equations for FemW-tail and trunk-tail relationships show slightly higher prediction errors than the PGLS versions. Among the three predictors examined, trunk length serves as the most effective variable for estimating tail length. Considering that future refinements to the phylogenetic framework may influence PGLS results, the OLS models for both trunk-rib (Eq. (9)) and trunk-tail (Eq. (15)) relationships were selected as the preferred estimators in this study.

For the pruned skull-neck dataset, the PGLS model also indicates a significant correlation between the skull-neck ratio and cervical number (*P* < 0.001). Among the three models evaluated, the nonlinear log–logistic model (LL; Eq. (18)) significantly outperforms both the PGLS (Eq. (16)) and OLS (Eq. (17)) models, as reflected in its lowest AICc and $\overline{ \left\vert \%\mathrm{PE} \right\vert }$ values.

(16) \begin{eqnarray*}\mathrm{PGLS:}{\log \nolimits }_{10} \left( \frac{\mathrm{SKL}}{\mathrm{SKL+neck}} \right) =-1.4695{\log \nolimits }_{10} \left( \mathrm{CN} \right) +1.5560 \mathrm{AICc}=-74.11\nonumber\\\displaystyle  \overline{ \left\vert \%\mathrm{PE} \right\vert }=19.00\end{eqnarray*}

(17) \begin{eqnarray*}\mathrm{OLS:}{\log \nolimits }_{10} \left( \frac{\mathrm{SKL}}{\mathrm{SKL+neck}} \right) =-1.4851{\log \nolimits }_{10} \left( \mathrm{CN} \right) +1.5568 \mathrm{AICc}=-69.10\nonumber\\\displaystyle  \overline{ \left\vert \%\mathrm{PE} \right\vert }=18.63\end{eqnarray*}

(18) \begin{eqnarray*}\mathrm{LL:}{\log \nolimits }_{10} \left( \frac{\mathrm{SKL}}{\mathrm{SKL+neck}} \right) = \frac{1.2808}{1+{ \left( \frac{{\log \nolimits }_{10} \left( \mathrm{CN} \right) }{1.5844} \right) }^{10.2843}} -1.4398 \mathrm{AICc}=-94.18\nonumber\\\displaystyle  \overline{ \left\vert \%\mathrm{PE} \right\vert }=11.92.\end{eqnarray*}

For the whole dataset, the nonlinear model still outperforms the OLS, thus it (Eq. (20)) was used in subsequent reconstructions due to its performance in both fit and prediction.

(19) \begin{eqnarray*}\mathrm{OLS:}{\log \nolimits }_{10} \left( \frac{\mathrm{SKL}}{\mathrm{SKL+neck}} \right) =-1.5065{\log \nolimits }_{10} \left( \mathrm{CN} \right) +1.5916 \mathrm{AICc}=-73.70\nonumber\\\displaystyle  \overline{ \left\vert \%\mathrm{PE} \right\vert }=19.07\end{eqnarray*}

(20) \begin{eqnarray*}\mathrm{LL:}{\log \nolimits }_{10} \left( \frac{\mathrm{SKL}}{\mathrm{SKL+neck}} \right) = \frac{1.3067}{1+{ \left( \frac{{\log \nolimits }_{10} \left( \mathrm{CN} \right) }{1.5937} \right) }^{10.2847}} -1.4684 \mathrm{AICc}=-103.58\nonumber\\\displaystyle  \overline{ \left\vert \%\mathrm{PE} \right\vert }=11.86.\end{eqnarray*}

### Plesiosaur models and mass equations

**Figure 5:**
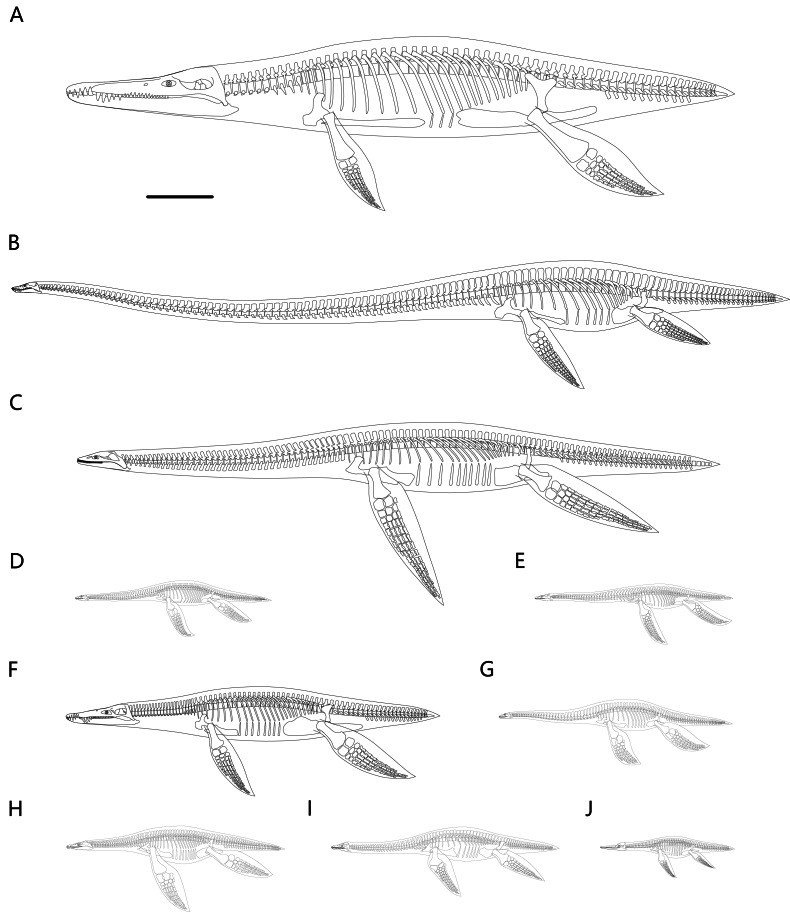
Representative plesiosaur reconstructions created in this study. (A) *Kronosaurus queenslandicus*. (B) *Albertonectes vanderveldei*. (C) *Aristonectes quiriquinensis*. (D) *Plesiopterys wildi*. (E) *Brancasaurus brancai*. (F) *Liopleurodon ferox*. (G) *Seeleyosaurus guilelmiimperatoris*. (H) *Meyerasaurus victor*. (I) *Cryptoclidus eurymerus*. (J) *Mauriciosaurus fernandezi*. All limbs are vertically oriented for display. The dorsal ribs in these skeletal reconstructions have been aesthetically refined and thus differ from the initial versions used to infer ribcage cross-sections (as shown in Figs. 1E and 3A). Scale bar equals 1 m.

Twenty-seven plesiosaur reconstructions were generated following the methodological criteria presented in this study. As shown in Fig. 5, the body sizes of some thalassophonean pliosaurs and elasmosaurids considerably exceed those of other plesiosaur clades. The estimated body lengths along the vertebral column and body masses are presented in Table 1.

**Table 1:** Estimated body lengths and masses of the 27 plesiosaur models. All models, along with the derived body length and mass estimates, were constructed and calculated using the methods proposed in this study. The body axes of the *Pliosaurus* cf. *kevani* and *Pliosaurus funkei* models were generated by combining elements from both specimens, while their flipper dimensions reflect the specific differences between them (see Supplementary Material). Independent models were created for all other species. Osteologically immature individuals are marked with an asterisk *.

Taxon	Catalogue number	Clade	Length (m)	Mass (kg)
*Albertonectes vanderveldei*	TMP 2007.011.0001	Elasmosauridae	12.117	3,107
*Aristonectes quiriquinensis*	SGO.PV.957	Elasmosauridae	10.000	4,166
*Hydrotherosaurus alexandrae*	UCMP 33912	Elasmosauridae	8.795	1,877
*Styxosaurus* sp.	SDSM 451	Elasmosauridae	11.276	3,080
*Thalassomedon haningtoni*	DMNH 1588	Elasmosauridae	11.953	6,791
*Vegasaurus molyi*	MLP 93-I-5-1	Elasmosauridae	6.151	839
*Abyssosaurus nataliae*	MChEIO PM/1	Cryptoclididae	6.739	1,238
*Cryptoclidus eurymerus*	NHMUK PV R2860	Cryptoclididae	3.542	360
*Dolichorhynchops osborni*	FHSM VP404	Polycotylidae	2.958	425
*Martinectes bonneri*	KUVP 40002	Polycotylidae	4.951	1,272
*Mauriciosaurus fernandezi* *	INAH CPC RFG 2544 P.F.1.	Polycotylidae	2.083	79
*Polycotylus latipinnis*	YPM 1125	Polycotylidae	5.074	1,039
*Kronosaurus queenslandicus*	MCZ 1285	Pliosauridae	10.445	11,396
*Liopleurodon ferox*	GPIT-RE-3184	Pliosauridae	5.828	1,795
*“Monquirasaurus” boyacensis*	MJACM 1	Pliosauridae	9.130	9,917
*Peloneustes philarchus*	GPIT-RE-3182	Pliosauridae	4.337	1,035
*Pliosaurus* cf. *kevani*	CAMSM J.35990	Pliosauridae	9.791	10,739
*Pliosaurus funkei*	PMO 214.135	Pliosauridae	9.791	10,893
*Sachicasaurus vitae*	MP111209-1	Pliosauridae	10.354	12,824
*Stenorhynchosaurus munozi* *	VL17052004-1	Pliosauridae	5.959	2,149
*Macroplata tenuiceps*	NHMUK PV R5488	Rhomaleosauridae	4.385	604
*Meyerasaurus victor*	SMNS 12478	Rhomaleosauridae	3.391	374
*Microcleidus tournemirensis*	MMM J. T. 86-100	Microcleididae	4.185	223
*Seeleyosaurus guilelmiimperatoris*	SMNS 12039	Microcleididae	3.658	199
*Brancasaurus brancai* *	GPMM A3.B4	Leptocleidia	3.072	157
*Nichollssaura borealis* *	TMP 1994.122.0001	Leptocleididae	2.708	118
*Plesiopterys wildi* *	MH 7	Plesiosauroidea	3.029	131

Table 2 summarizes the performance of various skeletal structures as body mass proxies under Ordinary Least Squares (OLS) regression, while Table 3 presents corresponding Phylogenetic Generalized Least Squares (PGLS) results based on pruned datasets (excluding *Styxosaurus* SDSM 451). All variables examined showed statistically significant correlations with estimated body mass(*P* < 0.001 in PGLS models). Among these, trunk length and the dorsal centrum volume(mean length × mean width × mean height) emerged as the most effective predictors, exhibiting high *R*^2^ values, low $\overline{ \left\vert \%\mathrm{PE} \right\vert }$ and $\mathrm{sd} \left( \left\vert \%\mathrm{PE} \right\vert \right) $ values (Table 2). Although centrum area (mean width × mean height) and centrum volume yield similar mean prediction errors, the former exhibits a standard deviation more than twice that of the latter (Table 2). This indicates that centrum area yields relatively unstable body mass predictions across different species. The OLS models for trunk and centrum volume substantially outperform their PGLS counterparts ($ \left\vert \Delta \mathrm{AICc} \right\vert > 10$, Table 3), and the versions in Table 2 are therefore recommended for predictive use.

**Table 2:** Parameters of the regression models based on Ordinary Least Squares. All continuous measurements are in meters (m), and all proxies and the masses were log_10_-transformed before analysis. The vertebral “volume” and “area” represent proxies for the volume and cross-sectional area of the dorsal vertebral centra (approximated by mean length × mean width × mean height, and mean width × mean height, respectively). The humerus or femur “width” represents maximum width at the distal end. See Fig. S1 for measurement criteria.

Proxy	Slope	Intercept	*N*	*P*-value	*R* ^2^	$\overline{ \left\vert \%\mathrm{PE} \right\vert }$	$\mathrm{sd} \left( \left\vert \%\mathrm{PE} \right\vert \right) $
SKL×CN	2.8228	−0.3800	19	<0.001	0.9169	50.76	43.57
Trunk	2.9292	2.4367	27	<0.001	0.9836	17.73	13.94
Vertebral volume	1.0257	6.5333	14	<0.001	0.9768	22.65	12.40
Vertebral area	1.4711	6.2890	14	<0.001	0.9657	25.83	25.16
Humerus length	2.9617	4.2510	26	<0.001	0.8314	60.67	79.48
Humerus width	3.4183	5.3925	26	<0.001	0.8230	63.76	67.39
Femur length	2.5684	4.0469	24	<0.001	0.8150	73.21	107.42
Femur width	3.1556	5.2276	24	<0.001	0.9055	43.24	47.89
Coracoid length	2.5219	3.7767	17	<0.001	0.9041	47.03	33.08
Coracoid width	2.7437	4.6120	24	<0.001	0.9144	40.64	31.44
Pubic length	2.3601	4.1001	19	<0.001	0.8559	53.51	53.45
Pubic width (max)	2.9436	4.4309	20	<0.001	0.9076	44.75	35.66
Ischium length	2.0943	3.9879	21	<0.001	0.8305	63.40	74.11
Ischium width	2.9946	4.8402	23	<0.001	0.9455	35.44	41.75

**Notes.**

Abbreviations N sample size $\overline{ \left\vert \%\mathrm{PE} \right\vert }$ mean per cent prediction error $\mathrm{sd} \left( \left\vert \%\mathrm{PE} \right\vert \right) $ standard deviation of per cent prediction error SKL skull length CN cervical number

**Table 3:** Parameters of the Phylogenetic Generalized Least Squares (PGLS) models and their comparison with corresponding Ordinary Least Squares (OLS) models, based on the pruned datasets (with *Styxosaurus* SDSM 451 removed). All continuous measurements are in meters (m), and all proxies and the masses were log_10_-transformed before analysis. Abbreviations: N, sample size; AICc, sample-size corrected Akaike Information Criterion; ΔAICc = AICc_PGLS_- AICc_OLS_; SKL, skull length; CN, cervical number; The vertebral “volume” and “area” represent proxies for the volume and cross-sectional area of the dorsal vertebral centra. The humerus or femur “width” represents maximum width at the distal end. See Fig. S1 for measurement criteria.

Proxy	Slope_PGLS_	Intercept_PGLS_	*N*	*P*-value	AICc_PGLS_	AICc_OLS_	ΔAICc
SKL×CN	4.1469	−1.8817	18	<0.001	28.69	0.67	28.01
Trunk	2.8160	2.4871	26	<0.001	−34.70	−46.94	12.24
Vertebral volume	0.9924	6.3991	13	<0.001	1.94	−17.82	19.76
Vertebral area	1.4067	6.1423	13	<0.001	5.74	−12.01	17.75
Humerus length	3.1155	4.1540	25	<0.001	30.76	9.29	21.47
Humerus width	3.0397	5.1027	25	<0.001	21.55	13.90	7.66
Femur length	3.3919	4.2695	23	<0.001	31.41	11.51	19.91
Femur width	2.6469	4.8335	23	<0.001	13.68	−2.26	15.94
Coracoid length	2.1043	3.6265	16	<0.001	8.1	0.15	7.95
Coracoid width	2.8824	4.6642	23	<0.001	−6.18	−8.22	2.04
Pubic length	2.2077	4.0294	18	<0.001	17.35	6.86	10.49
Pubic width (max)	3.0576	4.4702	19	<0.001	5.33	0.93	4.39
Ischium length	1.9700	3.8969	20	<0.001	19.48	12.25	7.23
Ischium width	3.0640	4.8734	22	<0.001	−0.19	−11.32	11.13

**Figure 6:**
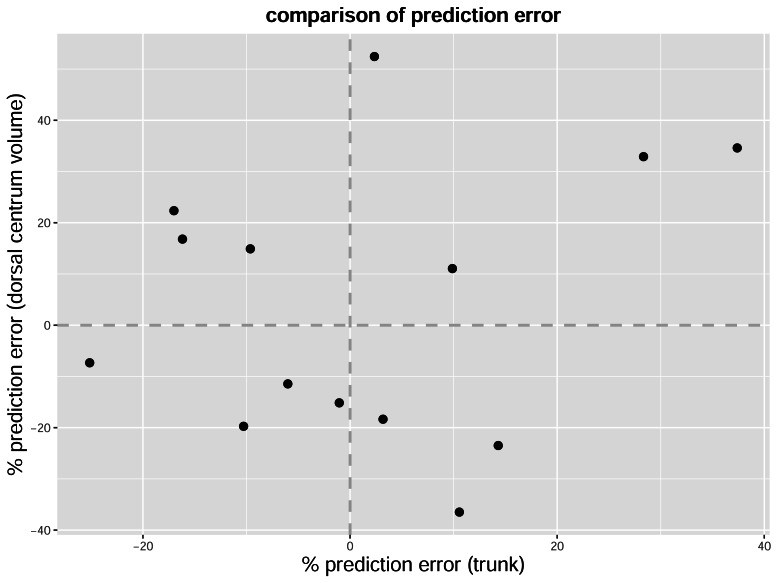
Scatter plot of prediction error for body mass estimates based on trunk length and dorsal centrum volume. The dorsal centrum volume represents mean length × mean width × mean height. Positive prediction error values indicate underestimation of body mass, while negative values indicate overestimation.

A scatter plot showing the prediction errors (without taking the absolute value) based on trunk length and dorsal centrum volume is presented in Fig. 6. The presence of data points in the second and fourth quadrants of the coordinate system indicates that the two formulae sometimes generate incongruent results when dealing with the same individual. That is, while dorsal centrum volume may overestimate the body mass in some cases, trunk length may underestimate it, and *vice versa*. The presence of such inconsistencies suggests that extra caution should be exercised when applying these body mass formulae (see Discussion).

## Discussion

### Selection of independent variables in the regression analyses

The selection of independent variables was based on a thorough evaluation of potential body modules and the availability of fossil measurement data. Although Knutsen, Druckenmiller & Hurum (2012) estimated skull length in some thalassophonean pliosaurs using the width of cervical vertebrae, this method is unlikely to be broadly applicable across Plesiosauria due to their highly variable cervical numbers and body proportions. Informed by the previously established knowledge that longer necks tend to associate with smaller heads in plesiosaurs (O’Keefe, 2002), this study hypothesized a negative correlation between cervical number and the skull-to-neck length ratio. The cervical number was treated as a pseudo-continuous variable, following O’Keefe (2002). The regression models confirmed this negative correlation as statistically significant (Eqs. (16)–(20)).

Both maximum rib arc length and trunk length are pivotal determinants of ribcage size and may represent different measurements of a potential body module (*i.e.,* the ribcage). In addition to this biological rationale, practical access to fossil measurements was also a key factor in variable selection. Specimens with relatively complete rib sequences often possess articulated trunk regions, enabling direct measurement of trunk length (*e.g.*, *Seeleyosaurus guilelmiimperatoris* SMNS 12039, Sachs et al., 2025; *Albertonectes vanderveldei* TMP 2007.011.0001, Kubo, Mitchell & Henderson, 2012). Theoretically, dorsal vertebral dimensions could also serve as a proxy for rib length due to their direct anatomical articulation. However, validation of this hypothesis remains constrained by limited sample availability. In many articulated specimens (*e.g.*, *“Monquirasaurus” boyacensis* MJACM 1, Noè & Gómez-Pérez, 2022; *Brachauchenius lucasi* USNM 4989, McHenry, 2009), the dorsal vertebrae are partially embedded in the matrix, hence it is impossible to obtain certain vertebral dimensions from them. Consequently, the available dataset of associated vertebral-rib measurements is limited and does not comprehensively represent the taxonomic diversity of Plesiosauria (*e.g.*, the absence of rhomaleosaurids and leptocleidids from this dataset).

**Figure 7:**
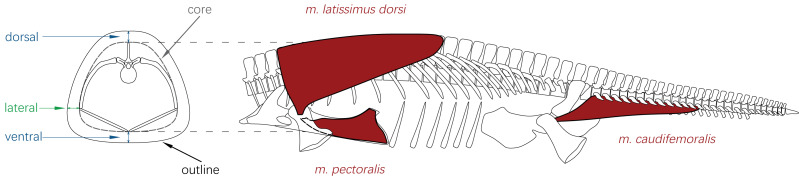
The glenoid cross-section and part of the *Cryptoclidus eurymerus* reconstruction in lateral view. The origin and insertion locations of the muscles mentioned in the text were reconstructed according to the description in Krahl & Witzel (2021). For more comprehensive illustrations of limb musculature of plesiosaurs, see Carpenter et al. (2010) and Araújo & Correia (2015).

Some previous studies have estimated total body length for plesiosaurs with incomplete caudal sequences (*e.g.*, Noè & Gómez-Pérez, 2022; Martill, Jacobs & Smith, 2023), but a standardized method for predicting tail length remains lacking. Although the potential functional role of the tail in propulsion has been discussed (Sennikov, 2019), no qualitative or quantitative correlations between tail length and other anatomical dimensions have been previously established. To address this gap, this study proposes and quantitatively evaluates two hypotheses concerning size correlations with tail length: (1) the trunk and tail, as interconnected segments of the body axis, may scale in a coordinated manner; (2) the anterior part of the tail and hindlimbs may function as an integrated musculoskeletal module. Muscle reconstructions in plesiosaurs suggest that one of the hindlimb retractors, *m. caudifemoralis*, originated on the tail, potentially spanning from the first to approximately the thirteenth caudal vertebra (Krahl & Witzel, 2021). *M. caudifemoralis* inserted on the proximal end of the femur, thereby creating a structural connection between the tail and the hindlimb (Fig. 7). Given the frequent incomplete preservation of distal limb elements (*e.g.*, phalanges) in plesiosaurs, femur length and distal width were selected as independent variables to test the second hypothesis.

This study did not evaluate the predictive performance of skull length or total body length for body mass estimation, owing to the considerable variation in neck length among plesiosaurs (Fig. 5). Multiplying the skull length (SKL) by cervical number (CN) may compensate for their individual weaknesses, creating a composite variable. This variable could be particularly useful for estimating mass in specimens preserving only the skull and neck (*e.g.*, *Thaumatodracon wiedenrothi* NLMH 106.058, Smith & Araújo, 2017). However, its predictive precision for body mass remains limited, as reflected in the high $\overline{ \left\vert \%\mathrm{PE} \right\vert }$ values (Table 2). Among the proxies tested, trunk length has been hypothesized in previous studies as a reliable indicator of plesiosaur body size (Benson, Evans & Druckenmiller, 2012; Gutarra et al., 2022). The regression models in this study support its validity (Tables 2 and 3). Extensive prior work has identified limb bone dimensions as effective predictors of body mass in terrestrial tetrapods, based on the biomechanical principle that greater mass requires more robust limb bones for support (Anderson, Hall-Martin & Russell, 1985; Campione et al., 2014; Campione & Evans, 2020). Since plesiosaurs were primarily propelled by their four flippers (Krahl, 2021), a similar logic may apply: larger species likely required larger appendages for effective propulsion. Therefore, this study assesses the utility of propodial (humerus and femur) length and distal width as mass indicators. The analysis was also extended to the massive pectoral and pelvic girdle bones, which served as anchor points for the muscles driving flipper movement (Carpenter et al., 2010; Araújo & Correia, 2015; Krahl & Witzel, 2021). The regression results revealed a statistically significant but relatively weak correlation between each limb or girdle variable and body mass (Table 2). This may be partially obscured by the coordinated evolution between neck length and locomotor apparatus size, as identified by O’Keefe (2002). Besides the limb dimensions, Romer & Price (1940) hypothesized that the combined dimensions of dorsal vertebrae, specifically the cross-sectional area approximated from centrum diameters, could indicate body mass by reflecting the load-bearing capacity of the spine. This hypothesis has been adopted in a recent study regarding the body sizes of Carboniferous synapsids (Brocklehurst & Brink, 2017), though its reliability has not yet been rigorously validated. In this study, the predictive performance of the proxies for the volume and cross-sectional area of dorsal vertebral centrum was tested. Regression results showed that both significantly outperformed skull length × cervical count (SKL × CN) as well as limb and girdle dimensions (Table 2).

### Non-linearity after log-transformation in skull-neck relationship

Linear regression models based on Ordinary Least Squares can effectively capture the covariation between two sets of linear measurements when they follow an isometric relationship (Draper & Smith, 1998). Biological systems, however, frequently exhibit power-law scaling patterns (Savage et al., 2004; Marquet et al., 2005; Schuster, 2010). Logarithmic transformation of such datasets can convert these nonlinear relationships into forms amenable to linear fitting. But the typical assumption that log-transformation completely linearizes the relationship does not always hold (Gayford et al., 2024). Some previous studies reported the potential systematic biases in prediction arising from this assumption, revealing that nonlinearity after log-transformation is not uncommon in biological scaling (Knell, 2009; Venditti, Baker & Barton, 2024). However, neither well-defined criteria for determining when to apply nonlinear regression nor the models incorporating phylogeny have been developed. Therefore, the application of nonlinear models relies entirely on empirical evaluation in the current stage.

**Figure 8:**
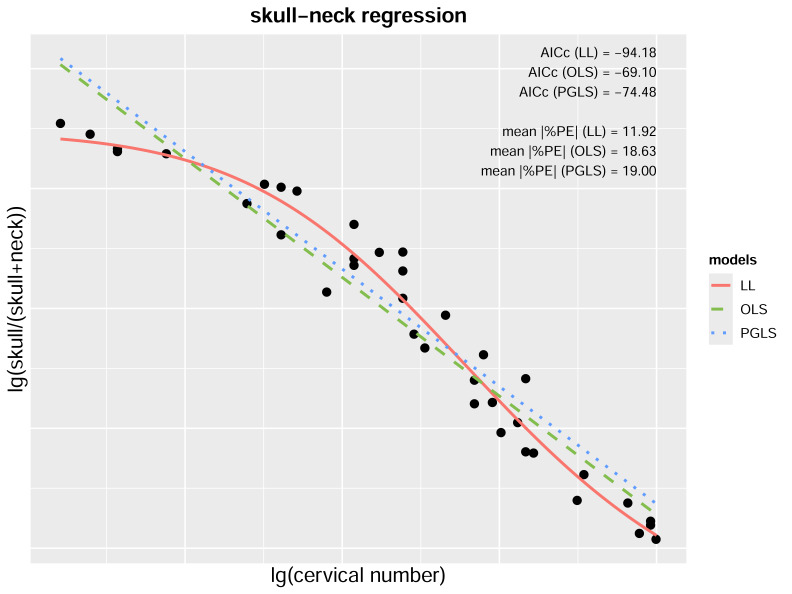
Scatter plot and regression models of the pruned skull-neck dataset. The red curve, green dashed line, and the blue dotted line represent the nonlinear regression model based on a log–logistic (LL) function, the Ordinary Least Squares (OLS) model, and the Phylogenetic Generalized Least Squares (PGLS) model, respectively. Lower values of sample-size corrected Akaike Information Criterion (AICc) and mean per cent prediction error ($\overline{ \left\vert \%\mathrm{PE} \right\vert }$) represent better model performance.

Figure 8 shows the distribution of sample points of the log-transformed skull-neck dataset. When a linear model is fitted, the data points at both ends of the horizontal axis tend to fall below the regression line, while those in the middle lie above the line, suggesting potential inadequacy of the linear fit. The distribution of scatter points visually resembles a logistic curve, hence the dataset was subjected to a nonlinear regression using a log–logistic function (Eqs. (18) and (20)). The AICc and $\overline{ \left\vert \%\mathrm{PE} \right\vert }$ values confirmed that this model yields better performance than the OLS or PGLS regression; it was therefore employed to estimate the skull lengths of plesiosaur models in this study. The scatter plots of all other regression models were also manually examined, and no nonlinear model after log-transformation was considered feasible for them.

### Uncertainty in soft tissue reconstruction of plesiosaurs

Soft tissue traces are preserved in a number of plesiosaur fossils, with the earliest records dating back to the 19th century (Sollas, 1881; Dames, 1895). Most plesiosaur fossils preserving soft tissues are plesiosauroids from Jurassic deposits in Germany, with remains typically surrounding the vertebral column or limbs (Vincent et al., 2017; Sachs et al., 2025; Marx et al., 2025). In contrast, *Mauriciosaurus fernandezi* is represented by a nearly complete specimen with extensive soft tissue traces surrounding the ribcage, revealing the *in vivo* body outline of plesiosaurs (Frey et al., 2017).

Nevertheless, the existence of these fossils does not fully resolve the challenges in plesiosaur reconstruction. To date, no fossil specimen preserves unambiguous evidence of the *in vivo* soft tissue contours in the head and neck regions. Although a more robust neck has been proposed to confer hydrodynamic benefits (Troelsen et al., 2019), quantitative criteria for reconstructing neck outlines remain lacking. In addition, no currently known plesiosaur fossils reveal soft contours along the leading edges of the flippers or the propodial regions, and the shape of the flipper *in vivo* was probably not a direct extension of the convex hull formed by the limb bones, based on evidence from modern aquatic tetrapods (DeBlois & Motani, 2019). In this study, only minimal soft tissue was added around the skull, following observations of extant aquatic reptiles (Wyneken, 2003; Holliday et al., 2022). It has been demonstrated that the limited craniofacial soft tissue in leatherback turtles (*Dermochelys coriacea*) is sufficient to maintain body heat in cold environments (Davenport et al., 2009), and a similar mechanism may also be present in plesiosaurs. The neck outline was reconstructed as a smooth curve connecting the skull and trunk, consistent with the morphology seen in modern aquatic tetrapods (Riedman, 1990; Huggenberger, Oelschläger & Cozzi, 2018). These reconstructions of head and neck soft tissue should be regarded as provisional and hypothetical, pending further evidence from future fossil discoveries and physiological studies. Referring to penguins or sea turtles represents a well-established approach in prior reconstructions of plesiosaur flippers (Muscutt, 2017; Henderson, 2024); accordingly, this study adopts the same approach in the absence of fossil specimens preserving complete soft-tissue outlines.

Gutarra et al. (2022) utilized *M. fernandezi* as a reference to quantitatively restore the soft tissue enveloping the plesiosaur ribcage. Despite the overall completeness of the specimen, its ribcage is fully collapsed, and all dorsal ribs are partially obscured by overlying girdle elements and gastralia (Frey et al., 2017). It therefore remains uncertain whether the fossil accurately reflects the ribcage width *in vivo*. Furthermore, habitat preference may also influence soft tissue volume: cetaceans in high-latitude environments typically possess thicker blubber than those in warmer waters (McLellan et al., 2002; Tang et al., 2023). Some plesiosaurs are known to have inhabited or seasonally migrated to high-latitude regions (Weems & Blodgett, 1996; Otero, Soto-Acuna & O’Keefe, 2018; Roberts et al., 2020), suggesting they may have carried more adipose tissue than *M. fernandezi*, which is interpreted as having inhabited warm and temperate environments (Frey et al., 2017; Topper et al., 2011). Since no quantitative framework is currently available to account for such ecological variation, the soft tissue contour of *M. fernandezi*, 25% wider than the ribcage (Frey et al., 2017), was adopted as a compromise criterion and applied uniformly across all plesiosaur models. However, caution is required as this value was derived solely from figures published by Frey et al. (2017), without first-hand examination under ultraviolet light.

The fossil of *M. fernandezi* preserves no direct evidence of soft tissue thickness along the dorsal or ventral aspects of the ribcage (Fig. 7), leaving this issue open to inference. In transverse cross-sections of the trunk region of most extant aquatic tetrapods (excluding sea turtles), the ribcage is surrounded by a layer of muscle and ligament (termed the “core” in relevant studies, see Castellini et al., 2009, for example). External to this core lie adipose tissue and skin. In these taxa, the overall trunk cross-sectional profile generally forms a uniform outward expansion of the core profile (*i.e.,* the body and core profiles share the same geometric shape but differ in scale), regardless of locomotor mode (*e.g.*, see Castellini et al., 2009 for pinnipeds; Ivančić, Solano & Smith, 2014 and Huggenberger, Oelschläger & Cozzi, 2018 for cetaceans; Field et al., 2012 for penguins). Assuming a comparable anatomical organization in plesiosaurs, their trunk cross-sectional outline can be reconstructed once the profile of the muscular core has been established (Fig. 7).

Multiple studies have previously reconstructed the musculature of plesiosaurs (Carpenter et al., 2010; Araújo & Correia, 2015; Krahl & Witzel, 2021). The primary forelimb elevator, *m. latissimus dorsi*, originates from the neural spines of the anterior dorsal vertebrae and inserts on the anterodorsal tuberosity of the humerus (Fig. 7). This configuration differs markedly from that in sea turtles and penguins. In sea turtles, the main locomotor muscles of the forelimbs gather on the pectoral girdle and plastron, with no direct connection to the vertebral column (Wyneken, 2003). In penguins, the principal wing elevator (*M. supracoracoideus*) lies on the ventral side of the thorax, a trait shared by other avians (Bannasch, 1994). Dorsal muscle volume was incorporated into the present plesiosaur reconstruction to reflect its likely influence on trunk contour (Fig. 7). Additionally, the ventral surfaces of both the pectoral and pelvic girdles in plesiosaurs serve as attachment points for multiple muscles involved in limb control (Krahl & Witzel, 2021). Consequently, robust musculature was also restored on the ventral aspects of both girdles, yielding substantial, rounded cross-sectional outlines.

Contour fat was present in the tail region of *M. fernandezi* in life, forming a streamlined body outline from the trunk to the tail tip (Frey et al., 2017). A pygostyle-like structure formed by fused terminal caudals is phylogenetically widespread among plesiosaurs (see Clark, O’Keefe & Slack, 2023 and references therein). While previous studies have interpreted this structure as potential evidence of a tail fluke, its orientation (horizontal or vertical) and shape remain unclear to date (Wilhelm & O’Keefe, 2010; Smith, 2013; Otero, Soto-Acuna & O’Keefe, 2018; Sachs et al., 2025). Given its small size in plesiosaurs, omitting a hypothetical tail fluke would have a negligible impact on total body mass estimates.

### Limitations of the modeling protocol and caveats for the mass equations

Major limitations of the hybrid approaches include inconsistent modeling standards across studies, inadequate taxonomic sampling, and small sample sizes (Campione & Evans, 2020). This study seeks to mitigate the impact of the first two limitations by implementing a standardized protocol for modeling multiple plesiosaur species. This modeling framework attempts to circumvent the common patterns of incompleteness in plesiosaur fossils by establishing regression equations (Eqs. (8)–(20)) and selecting closely related taxa as references. As demonstrated in the reconstruction workflow for *Pliosaurus* cf. *kevani* and *Pliosaurus funkei* (Supplementary Material), these tools enable fossil material lacking certain elements (*e.g.*, the tail) to be augmented into complete volumetric models, thereby permitting body mass estimation. However, the modeling workflow proposed here imposes high demands on vertebral formula and measurements, rendering it inapplicable to many fragmentary fossil specimens (*e.g.*, *Eromangasaurus australis*, Kear, 2007; *Lorrainosaurus keileni*, Sachs et al., 2023; *Boyacasaurus sumercei*, Benavides-Cabra et al., 2025). Restoring missing structures according to phylogenetically close relatives also inevitably carries the risk of overlooking interspecific morphological variation, as revealed by the differences in spinal curvature and rib orientation within Cryptoclididae (O’Keefe et al., 2011). In addition to the uncertainty arising from fossil incompleteness, taphonomic distortion could also introduce potential estimation bias. During the volumetric modeling, the original shape of distorted elements was reconstructed by referring to well-preserved relative species (*e.g.*, restoring the dorsoventral depth of crushed skulls). This method may inevitably introduce a degree of subjectivity. However, since the total body mass is determined by multiple anatomical components, the bias resulting from the restoration of local structures is likely to be significantly diluted in the overall mass estimation. This local uncertainty may also be masked or offset by the uncertainty associated with estimating missing elements. Therefore, this subjectivity should have a limited impact on the overall order of magnitude of the final mass estimates.

To address the issue of inadequate taxonomic sampling inherent in the hybrid approach, this study established volumetric models for a phylogenetically broad range of plesiosaur species wherever possible. The current sample set contains six elasmosaurids, two cryptoclidids, four polycotylids, eight pliosaurids, two rhomaleosaurids, two microcleidids, one leptocleidid, one basal leptocleidian, and *Plesiopterys wildi*, covering all major plesiosaur clades. On the other hand, the issue of small sample size remains unavoidable, as the number of models that can be constructed is strictly constrained by the preservation state of available fossil material. For example, at least one fully preserved and exposed coracoid or pubis is required to determine ribcage width and height, thereby excluding some otherwise largely complete fossils from modeling (*e.g.*, *Rhomaleosaurus cramptoni*, Fig. 3H).

Previous studies have cautioned that regression models show limited reliability when predicting values outside the sample range (Gayford et al., 2024). From the modest *Mauriciosaurus fernandezi* (which is an osteologically immature individual, Frey et al., 2017) to the gigantic *Sachicasaurus vitae*, the plesiosaur models created in this study range from 79 kg to 12,824 kg (Table 1), covering sufficient breadth to accommodate the size diversity across most plesiosaurs. It is also noteworthy that *M. fernandezi*, *Brancasaurus brancai*, *Nichollssaura borealis*, and *Plesiopterys wildi* are excluded from the dorsal vertebrae datasets, as at least one vertebral dimension is unavailable due to preservation status or mounting (see Frey et al., 2017; Sachs, Hornung & Kear, 2016; Druckenmiller & Russell, 2008; Marx et al., 2025). The smallest sized specimen in the dorsal centrum volume-body mass dataset is *Seeleyosaurus guilelmiimperatoris* SMNS 12039, which remains smaller than most known plesiosaurs. On the other hand, fragmentary fossils of several pliosaur individuals from the Late Jurassic of Europe, previously assigned to the genus *Pliosaurus*, indicate body sizes larger than that of *S. vitae* (Tarlo, 1959; Martill, Jacobs & Smith, 2023). Investigation of their body sizes not only delineates the applicable boundaries of the mass equations, but also enables exploration of the potential maximum body size attained by plesiosaurs. The fossil materials of these pliosaur individuals indicate that they possibly reached or exceeded 20 metric tons in life (see Supplementary Material for a brief body size revision of the individual OUMNH PAL-J.010454), therefore caution is recommended when extrapolating the mass equations to them.

The volumetric models and regression analyses in this study demonstrate that trunk length and dorsal centrum volume are strongly correlated with body mass in plesiosaurs (Table 2). The low mean and standard deviation of prediction errors further indicate that both metrics perform reliably in body mass estimation. Consequently, the regression equations based on these two variables can be applied to many other plesiosaur fossils, enabling body mass estimates from trunk length and dimensions of dorsal vertebrae. One potential limitation is that the regression equations based on these two variables may inevitably lead to disparate results in some cases, as shown in Fig. 6. Given this uncertainty, future researchers are recommended to apply both equations in tandem to bracket a plausible body mass range and complement these approaches with volumetric modeling when fossil material permits.

### Future studies

The ancestral state of dorsal rib design in amniotes was probably bicapitate, articulating *via* a tuberculum and capitulum with the diapophysis and parapophysis, respectively (Cieri et al., 2020). This condition persists in most descendant lineages, whereas some clades (*e.g.*, plesiosaurs, squamates) evolved single-headed dorsal ribs that articulate solely with the corresponding diapophysis (Romer, 1956). Given that either type of articulation pattern is present in most amniotes, the method introduced here—inferring rib spatial orientation from vertebral morphology—can potentially be applied to other extinct clades. A potential limitation of this method is that the ribcages of most amniotes are dynamic structures: they utilize costal movement to ventilate their lungs, whereby the ribs undergo periodic rotation synchronized with the respiratory cycle (Brainerd & Owerkowicz, 2006). Nevertheless, at least one static model of the ribcage can still be inferred from the vertebral morphology, therefore this methodology holds promise for broader application in extinct amniotes.

A key assumption underlying the present workflow, however, is that ribs can be reasonably simplified as 2D structures. This condition does not hold for all extinct taxa; for example, the posterior dorsal ribs of sauropod dinosaurs exhibit distinct three-dimensional curvature (Taylor & Wedel, 2023), necessitating either full 3D modeling or more complex mathematical treatment. Moreover, the current modeling process depends substantially on manual operation within CAD software such as AutoCAD and can be time-intensive. The mathematical framework was specifically designed to interface with CAD environments, though rib rotation could also be implemented using alternative approaches such as quaternions or rotation matrices. Consequently, the entire modeling workflow, as well as the cross-sectional method, holds potential for automation within an open-source platform.

Beyond the plesiosaur models developed in this study, some additional species are represented by relatively complete fossil material. For instance, specimens of *Muraenosaurus leedsii* (Andrews, 1910) and *Simolestes vorax* (Andrews, 1913) from the Oxford Clay Formation appear to be suitable for modeling. However, the current author failed to obtain access to the relevant material during the preparation of this study despite attempts. Furthermore, detailed skeletal descriptions of *Franconiasaurus brevispinus* and *Microcleidus* spp. are currently in preparation (Sachs, Eggmaier & Madzia, 2024; Sachs et al., 2025), which would enable modeling of these highly complete specimens. In light of this, the present study does not restrict its scope to the currently available plesiosaur models, but instead establishes an open-source plesiosaur reconstruction repository (as part of *Mundus Cyclus* (https://github.com/Pliosaurus-kevani/Mundus-Cyclus), a program for precise skeletal modeling of extinct vertebrates), to which future reconstructions by the author will be added to supplement the existing dataset.

This study recognizes trunk length and dorsal centrum volume as robust predictors of body mass in plesiosaurs, enabling rapid and straightforward estimation of body mass for many plesiosaur taxa. The mass equations based on these two variables (Table 2) can support various subsequent applications, such as quantifying size allometry in form-function studies and investigating patterns of body size evolution through deep time. Furthermore, the utility of dorsal vertebral dimensions as reliable mass proxies in other extinct vertebrate clades, as initially proposed by Romer & Price (1940), represents a promising avenue for future investigation.

## Conclusions

This study aims to establish a hybrid methodological framework for estimating body mass in plesiosaurs by proposing a standardized protocol for skeletal reconstruction, applying it across a phylogenetically broad sample of plesiosaur taxa, and using the resulting models to identify anatomical proxies that reliably predict body mass. To this end, 27 plesiosaur models were constructed, with the dimensions of missing anatomical elements restored by regression equations and morphological data from closely related taxa. Regression analyses based on these volumetrically derived body masses reveal that trunk length and dorsal centrum volume are strongly correlated with body mass and yield low prediction errors, indicating high reliability as mass predictors. The model sample encompasses representatives from all major plesiosaur clades, supporting the applicability of these two mass equations across Plesiosauria. The resulting formulae enable future studies to rapidly and efficiently estimate plesiosaur body mass from trunk length or dorsal vertebrae, thereby facilitating downstream biomechanical and macroevolutionary analyses.

## Supplemental Information

10.7717/peerj.21146/supp-1 Supplemental Information 1 Detailed protocol and software commands for plesiosaur reconstruction

## Anatomical (including those in Supplementary Material):

BCL: basal condylar length (skull length from snout tip to the occipital condyle)
CoraW: coracoid width
CN: cervical number
FemL: femur length
FemW: femur distal width
ForeL: forelimb length
HindL: hindlimb length
HumL: humerus length
HumW: humerus distal width
PubW_max_: maximum pubic width
PubWA: pubic width at the acetabulum
RAL: rib arc length
RPH: rib plane height
RPW: rib plane width
SH: skull height
SKL: skull length (from snout tip to the quadrate)
SW: skull width

## Methodological

AICc: sample-size corrected Akaike Information Criterion
CSM: cross-sectional method
ES: extant scaling approaches
LL: log–logistic model
N: sample size
OLS: Ordinary Least Squares
OTU: operational taxonomic unit
—%PE—: per cent prediction error
$\overline{ \left\vert \%\mathbf{PE} \right\vert }$: mean prediction error
sd—%PE—: standard deviation of the prediction error
PGLS: Phylogenetic Generalized Least Squares
VD: volumetric-density approaches

## Institutional

CAMSM: Sedgwick Museum of Geology, Cambridge, U.K.
DMNH: Denver Museum of Nature and Science, Denver, U.S.A.
FHSM: Fort Hays State University, Sternberg Museum of Natural History, U.S.A.
GPIT: Geologisch-Paläontologisches Institut Tübingen, Tübingen, Germany
GPMM: Geomuseum der Universität Münster, Münster in Westfalen, Germany
INAH: Instituto Nacional de Antropología e Historia, Saltillo, Mexico
KK: Kronosaurus Korner, Richmond, Queensland, Australia
KUVP: Natural History Museum, University of Kansas, Lawrence, U.S.A.
LACM: Natural History Museum of Los Angeles County, California, U.S.A.
MB: Museum für Naturkunde Berlin, Berlin, Germany
MChEIO: Museum of Chuvash Natural Historical Society, Chuvashia, Russia
MCZ: Museum of Comparative Zoology, Harvard University, U.S.A.
MH: Urwelt-Museum Hauff, Holzmaden, Germany
MJACM: Museo El Fósil, Vereda Monquirá, Colombia
MLP: Museo de La Plata, Buenos Aires, Argentina
MMM: Museé Municipal de Millau
NHMUK: Natural History Museum, London, U.K.
NLMH: Niedersächsisches Landesmuseum, Hannover
OUMNH: Oxford University Museum of Natural History, Oxford, U.K.
PMO: Palaeontology Museum, Natural History Museum, Oslo, Norway
QM: Queensland Museum, Brisbane, Queensland, Australia
SDSM: South Dakota School of Mines, Rapid City, U.S.A.
SGO.PV: Museo Nacional de Historia Natural, Santiago, Chile
SMNS: Staatliches Museum für Naturkunde Stuttgart, Stuttgart, Germany
TMP: Tyrrell Museum of Palaeontology, Drumheller, Alberta, Canada
UCMP: Museum of Paleontology, University of California at Berkeley, Berkeley, California
USNM: National Museum of Natural History, Washington, D.C., U.S.A.
YPM: Yale Peabody Museum, New Haven, U.S.A.

## Additional Information and Declarations

### Data Availability

The following information was supplied regarding data availability: The code, data, and references for data are available at Zenodo and GitHub: - Zhao, R. (2026). plesiosaur-reconstruction (v2.1). Zenodo. https://zenodo.org/records/18458089

https://github.com/Pliosaurus-kevani/plesiosaur-reconstruction
All the plesiosaur models are available at Zenodo and GitHub: - Ruizhe Zhao. (2026). Mundus-Cyclus (Horizon 3). Zenodo. https://zenodo.org/records/18393429
- https://github.com/Pliosaurus-kevani/Mundus-Cyclus.
