# Supplementary material for: Body reconstruction and size estimation of plesiosaurs
Source: PeerJ. 2026 Apr 14;14:e21146. doi: 10.7717/peerj.21146 (PMC13089226; doi:10.7717/peerj.21146)
Supplement: Supplemental Information 1 [file peerj-14-21146-s001.pdf]

Body reconstruction and size estimation of plesiosaurs
Supplementary Material

Ruizhe Jackevan Zhao

JackevanChaos@outlook.com

Contents

1 Extra quantitative criteria **1**
1.1 Intervertebral distance and skull length 1
1.2 Spinal curvature and rib coefficient 2

2 Worked example: *Sachicasaurus vitae* **3**
2.1 Skull and vertebral column 3
2.2 Ribcage and body shape 5
2.3 Flippers and soft tissues 7

3 Worked examples: *Pliosaurus* cf. *kevani* and *Pliosaurus funkei* **8**
3.1 Body length 8
3.2 Ribcage and limbs 9

4 On some giant Jurassic pliosaurs **10**

5 Supplementary Table **10**

1 Extra quantitative criteria

The measurement criteria used in this study are illustrated in Figure S1.

1.1 Intervertebral distance and skull length

The dimensions of intervertebral cartilage in the cervical region vary among plesiosaur clades and correlate negatively with cervical count. Relative spacing values for major clades were derived from specimens with articulated cervical series and applied as reference standards in reconstructions: 13% for rhomaleosaurids, as in *Rhomaleosaurus thorntoni* [1]; 25% for thalassophonean pliosaurs, as in *Sachicasaurus vitae* [2] and *Brachauchenius lucasi* [3]; 8% for elasmosaurids, as in *Libonectes morgani* [4]; 9% for Late Jurassic and Early Cretaceous cryptoclidids, as in *Ophthalmothule cryostea* [5]; 13% for polycotylids, as in *Mauriciosaurus fernandezi* [6]; 7% for microcleidids, as in *Seeleyosaurus*

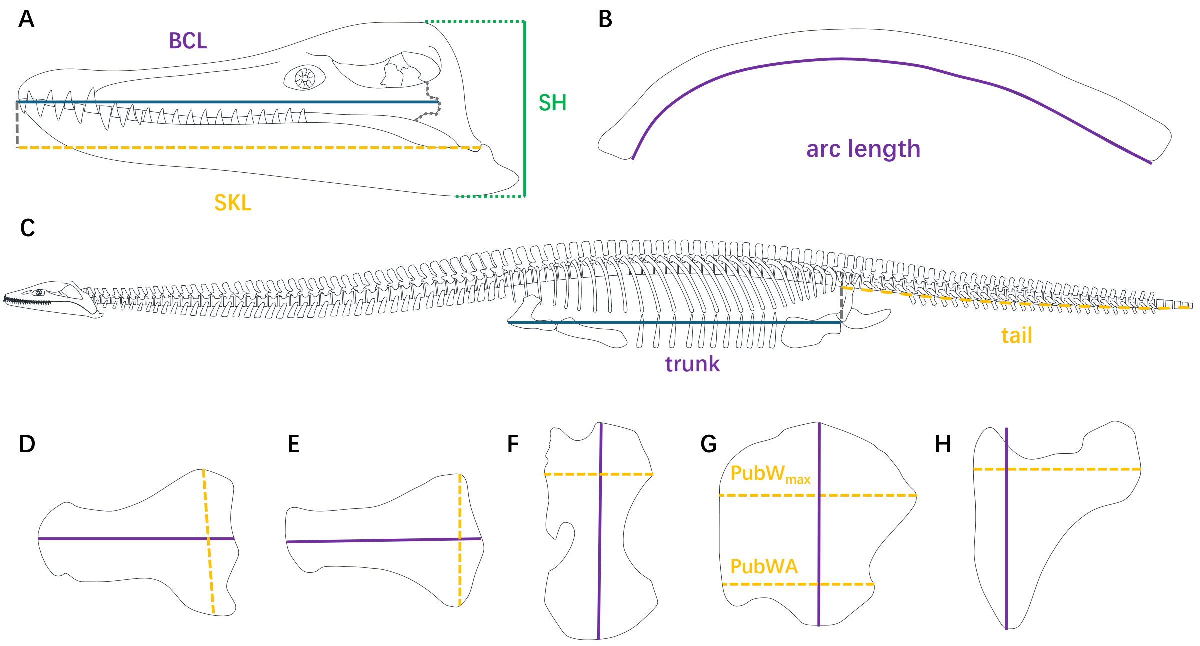

Figure 1: **Measurement criteria used in this study.** Criteria for (A) skull; (B) rib; (C) trunk and tail (D) humerus; (E) femur; (F) coracoid; (G) pubis; (H) ischium. Abbreviations: BCL, basal condylar length (from snout tip to occipital condyle); SH, skull height; SKL, skull length (from snout tip to quadrate); $PubW_{max}$, maximum pubic width; PubWA, pubic width at the acetabulum.

guilelmiimperatoris [7]. In contrast, intervertebral cartilage in the pectoral, dorsal, and sacral regions consistently accounts for approximately 10% of centrum length, as documented in multiple articulated specimens (e.g., *B. lucasi* [3], *R. thorntoni* [1], *Elasmosaurus platyurus* [8]). For specimens with incompletely preserved caudal series, tail length was estimated using the trunk-tail regression equation. In cases where the caudal vertebral series is nearly complete but not preserved in articulation, intervertebral spacing was reconstructed based on closely related taxa (see Supplementary Table for details). For specimens lacking skulls, skull length (SKL) was estimated via the skull-neck equation. Where possible, an alternative estimate was derived from the SKL-to-neck ratio of a congeneric species, with the final SKL taken as the mean of both values. Basal condylar length (BCL; Fig. S1A), measured from the snout tip to the occipital condyle, was subsequently calculated based on the SKL/BCL ratio of a closely related taxon (see Supplementary Table for details).

1.2 Spinal curvature and rib coefficient

For specimens with disarticulated vertebral columns, spinal curvature was reconstructed based on closely related taxa (*Rhomaleosaurus thorntoni* [1] for rhomaleosaurids; *Sachicasaurus vitae* [2] for thalassophonean pliosaurs; *Albertonectes vanderveldei* [9] for elasmosaurids; *Cryptoclidus eurymerus* [10] for cryptoclidids; *Dolichorhynchops osborni* [11] for polycotylids; *Seeleyosaurus guilelmiimperatoris* [7] for microcleidids).

For specimens lacking rib length data, the maximum rib arc length was estimated using the trunk-rib regression equation. The arc length of the rib dorsal to the glenoid was then reconstructed based

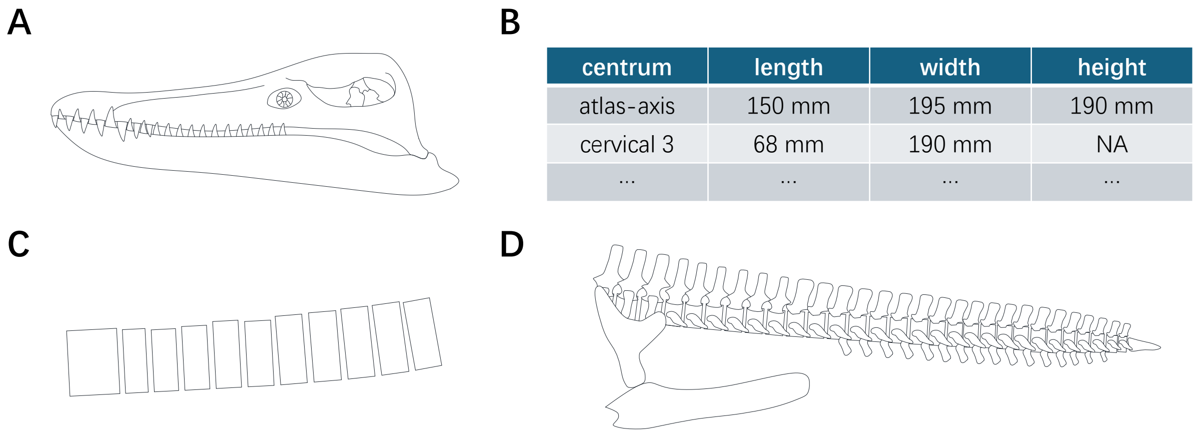

centrum	length	width	height
atlas-axis	150 mm	195 mm	190 mm
cervical 3	68 mm	190 mm	NA
...	...	...	...

Figure 2: **CAD line reconstructions of the skull and vertebrae of *Sachicasaurus vitae*.** (A) Line restoration of the skull of *S. vitae* in lateral view. (B) The dimensions of vertebral centra summarized in a table for later reference. (C) Line restoration of the cervical centra of *S. vitae*. Each centrum was represented by a rectangle with side lengths corresponding to its centrum length and height. Other vertebral centra can be reconstructed in the same way. (D) The reconstructed sacral and caudal vertebrae of *S. vitae*, together with the left ischium and ilium. The caudal count is hypothetical. Elements in different subfigures are not to scale.

on the rib coefficient (see the main text for definition) of a close relative (60% for rhomaleosaurids, as in *Rhomaleosaurus cramptoni* [12]; 80% for thalassophonean pliosaurs, as in *"Monquirasaurus" boyacensis* [13]; 66% for elasmosaurids, as in *Wapuskanectes betsynichollsae* [14]; 70% for polycotylids, as in *Dolichorhynchops osborni* [15]; 68% for cryptoclidids, based on pers. obs from an open source 3D scan of *Cryptoclidus eurymerus* IGPB R 324, from https://sketchfab.com/bcdh; 72% for microcleidids, as in *Microcleidus homalospondylus* [16]).

2 Worked example: *Sachicasaurus vitae*

This section details the specific software commands and operational procedures used throughout the modeling process. Using *Sachicasaurus vitae* as a representative example, it offers a step-by-step tutorial to allow readers to fully reproduce the modeling workflow described in this study, serving as a practical supplement to the main text.

2.1 Skull and vertebral column

The skull reconstruction of *S. vitae* in lateral view was derived from McHenry's reconstruction of *Kronosaurus queenslandicus* [3], but underwent extensive modifications (especially to the quadrate and dentition) to reflect its unique morphology (Fig. S2A). Skull height was reconstructed with reference to *Peloneustes philarchus* NHMUK PV R4058, which possesses a well-preserved cranium [17]; the snout proportions and mandibular depth of *S. vitae* itself were also taken into account [2]. In AutoCAD, restoring the line art of the skull requires the use of the *SPLINE* command to draw curves and the *TRIM* command to remove excess edges. The specific workflow is similar to that of digital

illustration in other software.

To reconstruct the vertebral column, the precaudal formula for the target taxon was first established. For example, *S. vitae* possessed 12 cervical, 2 pectoral, 23 dorsal, and 3 sacral vertebrae [2]. Where preservation permits, the length, height, and width of each vertebral centrum were summarized in a table (Fig. S2B). The lateral view of each vertebral centrum was approximated using a rectangle in AutoCAD, with side lengths equal to the centrum's length and height (Fig. S2C). This was achieved using the *RECTANG* command in AutoCAD. Other vertebral structures such as the neural arches and chevrons were drawn using *SPLINE*. In the holotype of *S. vitae*, centrum dimensions are documented in [2]. Within the dorsal vertebral sequence, only vertebrae D5, D13, D14, D15, D18, and D22 are preserved in a condition that permits accurate dimensional measurement [2]. Consequently, the mean centrum length of measurable dorsal vertebrae in *S. vitae* was compared with that of *Brachauchenius lucasi* USNM 4989, which has a complete and measurable dorsal series (see [3] for data), and the sum of dorsal lengths in *S. vitae* was calculated using the following formula (where *S.* denotes *Sachicasaurus* and *B.* denotes *Brachauchenius*):

$$\begin{aligned}\sum \text{length}_{S.} &= \frac{\text{mean length}_{S.}}{\text{mean length}_{B.}} \times \frac{\text{dorsal count}_{S.}}{\text{dorsal count}_{B.}} \times \sum \text{length}_{B.} \\ &= \frac{137.5\text{ mm}}{73.5263\text{ mm}} \times \frac{23}{19} \times 1397\text{ mm} \\ &= 3162.5\text{ mm}\end{aligned} \tag{1}$$

The sum of pectoral vertebral length was calculated in the same way. The trunk length was directly derived from fossil photographs using the photogrammetric method [2]. The corresponding axial length—the summed lengths of the pectoral and dorsal vertebrae plus the first sacral centrum, plus an additional 10% to account for intervertebral cartilage—was then calculated.

The method for reconstructing spinal curvature in the ribcage region has been described in the main text (see Figure 2 of the main text); therefore, only the relevant software commands are provided here. The horizontal line segment representing the trunk length was created using the *LINE* command, and the smooth curve representing the spinal curvature was created using the *SPLINE* command. The control points of the curve were adjusted manually to achieve the target length (i.e., combined length of the pectoral, dorsal, and the first sacral vertebrae). The *LIST* command was used intermittently to query and verify the current length of the curve during this process. Subsequently, a polyline was employed to approximate the curve using the *LINE* command. The previously created vertebrae were then aligned to this polyline using *ALIGN*, thereby completing the reconstruction of the spinal curvature within the ribcage. Finally, the neural arches were drawn using the *SPLINE* command.

The caudal series of *S. vitae* is incompletely preserved, hence the tail length was estimated using the trunk-tail equation. The modeling protocol for individual caudal centra is identical to that used for the presacral vertebrae. The current skeletal reconstruction comprises 26 free caudal vertebrae plus a terminal "pygostyle" fused by several centra (Fig. S2D). The latter is widespread across the Plesiosauria (see [18] for a review). Although the caudal count is entirely hypothetical, it falls within the observed range for complete or nearly complete plesiosaurian caudal counts in the fossil record (e.g., 28 in *Peloneustes philarchus* NHMUK PV R3318 [19]; >33 in *Albertonectes vanderveldei* TMP 2007.011.0001. [9]).

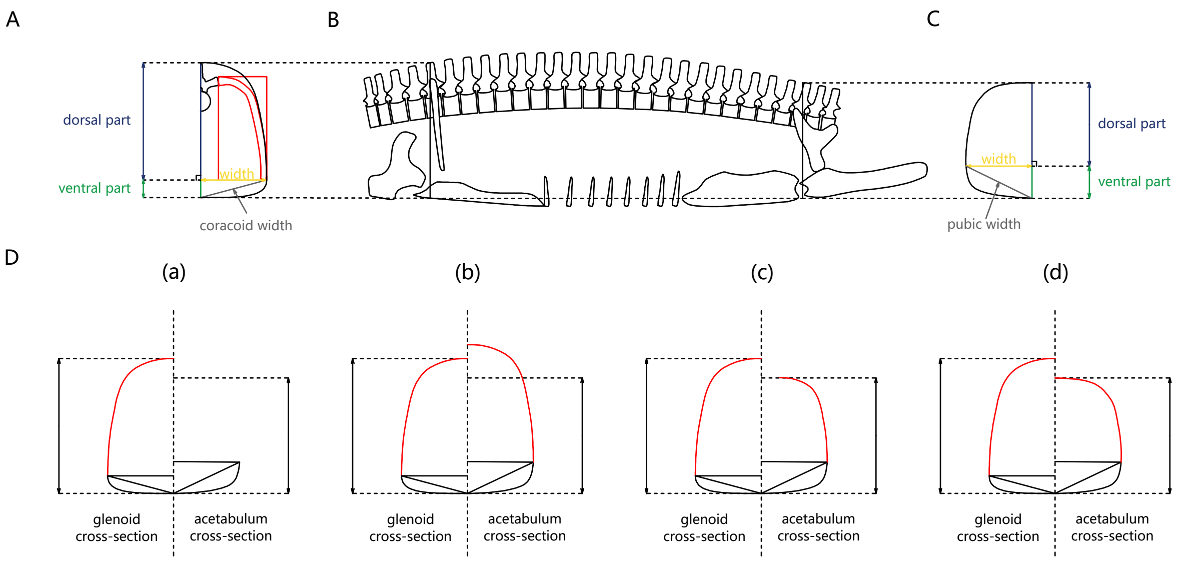

Figure 3: **CAD digital reconstructions showing steps to restore the glenoid and acetabulum cross-sections**. (A) The compositional elements of the glenoid cross-section. The red rectangle represents the rib plane projected to the front view. (B) Part of the restored ribcage of *Sachicasaurus vitae*, showing the positions of the glenoid and acetabulum cross-sections. (C) The compositional elements of the acetabulum cross-section. (D) Steps for reconstructing the dorsal outline of the acetabulum cross-section. See the text for detailed reconstruction procedure.

2.2 Ribcage and body shape

As noted in the main text, the first cross-section is positioned at the quadrate level. An ellipse was adopted to approximate the cross-sectional profile, with its major and minor axes corresponding to the maximum width and height (the latter remains hypothetical for *S. vitae*) of the skull, respectively.

In *S. vitae*, the ribs dorsal to the glenoid cavity are obscured by other ribs [2], thus the length of them was estimated by scaling the maximum rib arc length of *S. vitae* using the rib coefficient of *"Monquirasaurus" boyacensis* [13]. The longest rib of *S. vitae* has broken into two pieces [2], hence the maximum rib length was calculated by adding the arc lengths of them. As mentioned in the main text, only angle θ_3 requires consideration when reconstructing the glenoid cross-section. The corresponding rib plane was first reoriented in the 3D modeling environment of AutoCAD using the *ROTATE3D* command, then its frontal projection was aligned with the planar profile of the vertebral transverse process (Fig. S3A). A contour curve was subsequently traced along the outer margin of the rib silhouette in this view using the *SPLINE* command. In *S. vitae*, the pectoral girdle is obscured by the overlying ribs and vertebral column [2]. Consequently, the coracoid width was estimated by applying the pubis-to-coracoid width ratio of *Kronosaurus queenslandicus* MCZ 1285 [20]. The external contour of the ventral part was also drawn using the *SPLINE* command, adapting the reconstructions of preserved gastralia in other plesiosaurs (see the Materials and Methods section of the main text for references).

The ventral margins of both the glenoid and acetabulum cross-sections were assumed to lie within a common horizontal line, and the two sections were assigned an identical width (Figs. S3ABC). The

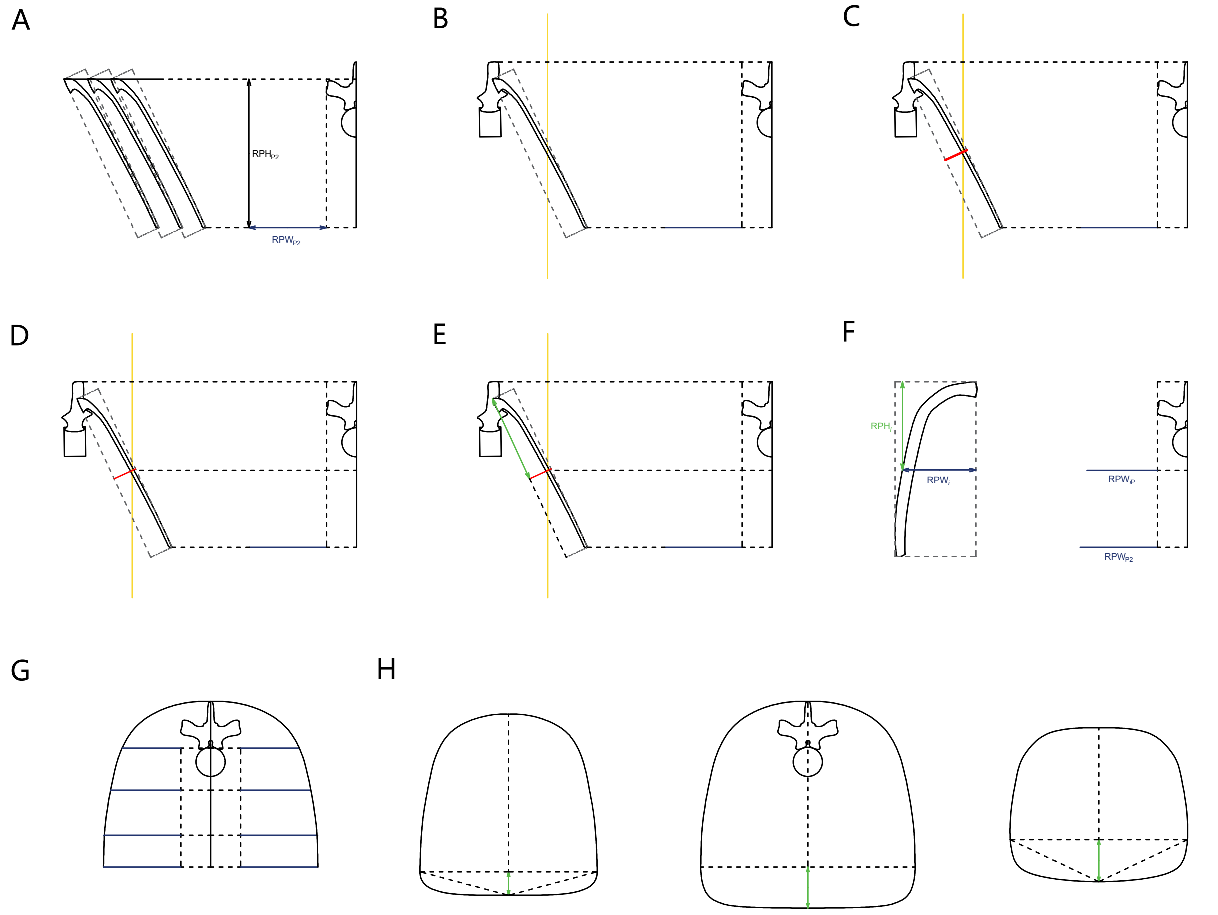

Figure 4: **CAD digital reconstructions showing steps to restore the middle cross-section**. (A) The rotated rib planes in lateral view, which were placed in the same horizontal level. (B)-(G) Steps to reconstruct the dorsal outline of the middle cross-section. The yellow vertical line represents the middle cross-section. See the text for description. (H) Derivation of the ventral height for the middle cross-section based on the corresponding values from the glenoid and acetabulum cross-sections.

total height of the acetabulum cross-section was derived from the ribcage reconstruction in lateral view (Figs. S3BC). Given the tendency of posterior dorsal ribs in plesiosaurs to become shortened and distally pointed [11], the contour of the acetabulum cross-section was derived through modification of the glenoid cross-section profile. Figure S3D shows the stepwise process: (1) the dorsal contour of the glenoid cross-section was initially replicated onto the acetabulum cross-section (Fig. S3D [b]); (2) this contour was then scaled using the *SCALE* command, adjusting its vertical dimension until the total cross-section height matched the value inferred from the lateral reconstruction of the ribcage (Fig. S3D [c]; note that the upper terminus of the curve remains unattached to the midline at this stage); (3) finally, the curve's dorsal endpoint was manually repositioned to meet the midline of the cross-section (Fig. S3D [d]). Similar to the glenoid cross-section, the external contour of the ventral part was established through modification to the reconstructed gastralia outlines.

To reconstruct the middle cross-section, the ribs intersected by this plane were assumed to lie at a uniform dorsoventral level and were assigned a standardized size equivalent to the maximum rib arc length (Fig. S4A). The rib plane was first rotated in 3D space using *ROTATE3D* within AutoCAD, and

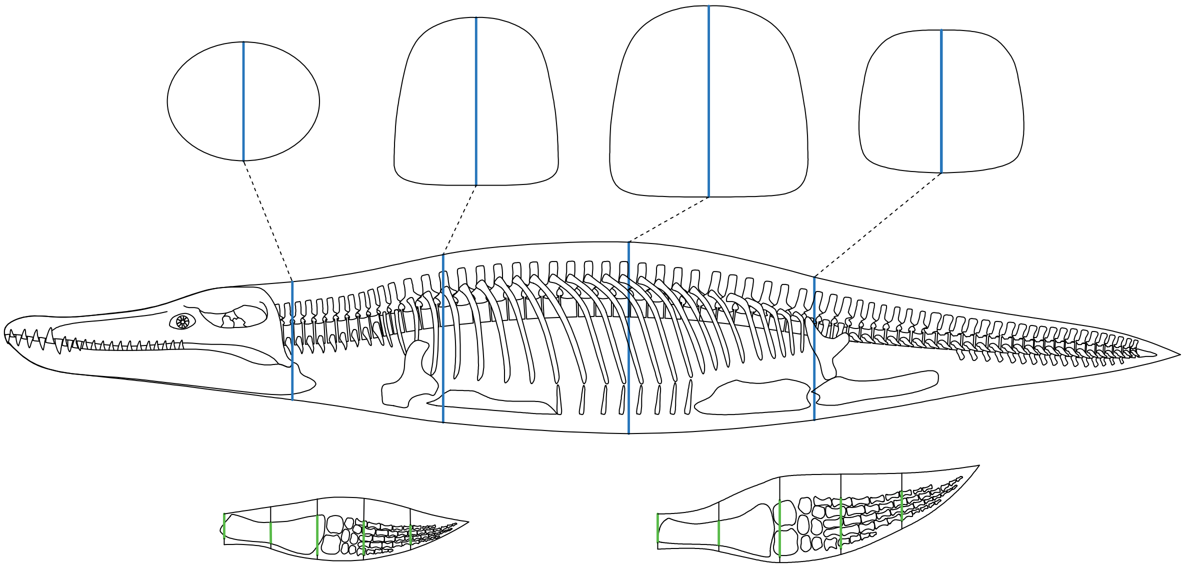

Figure 5: **The skeletal reconstruction of *Sachicasaurus vitae* constructed using AutoCAD, showing the restored cross-sections and soft outlines.**

its projected width and height in the frontal view were calculated using Equation (2) provided in the main text. For each rib intersected by the section plane (Fig. S4B), its positional coordinates within the cross-section are determined through a sequence of geometric computations. Figure S4 shows the stepwise derivation procedure for each of the intersected ribs: (1) the line segment representing rib plane width (RPW), corresponding to the upper or lower edge of the rib plane's bounding rectangle, was duplicated and repositioned to coincide with the intersection between the middle cross-section and the rib in lateral view (Fig. S4C); (2) the dorsoventral position of this intersection point, as projected onto the frontal view, was recorded (Fig. S4D); (3) the linear distance from this intersection to the dorsal margin of the rib plane was measured using the *MEASUREGEOM* command (Fig. S4E); (4) the measured value obtained in (3) was then used to locate the corresponding intersection point within the rib plane before rotation (i.e., where $\theta_1 = \theta_2 = \theta_3 = 0°$; Fig. S4F); (5) the horizontal distance (projected to the frontal view) between the intersection point and the vertebral transverse process was computed using the equation $\mathrm{RPW}_{iP} = \mathrm{RPW}_i \cdot \cos\theta_2$ (provided in the main text). Once the positions of all intersected ribs were established, a smooth curve was drawn through these points to complete the reconstruction of the dorsal outline (Fig. S4G). The height of the ventral part was calculated by dividing the average ventral height of the glenoid and acetabulum cross-sections by the rib coefficient (Fig. S4H; see Equation [4] in the main text).

2.3 Flippers and soft tissues

The distal elements of the limbs of *S. vitae* are poorly preserved, hence the total length of each limb was first estimated from its propodial distal width using the equation provided in [21]. Then the missing elements were restored according to those of *"Monquirasaurus" boyacensis* [13] using the *SPLINE* command. The soft outlines of the flippers were quantitatively restored according to the criteria proposed in [22]. For the body outline, the three ribcage cross-sections were enlarged by

25%, and smooth curves were created using *SPLINE* to connect their endpoints (Fig. 5).

3 Worked examples: *Pliosaurus* cf. *kevani* and *Pliosaurus funkei*

To demonstrate the application of the modeling workflow to more fragmentary fossil specimens, this section outlines the methodology employed to reconstruct the missing anatomical information for the *Pliosaurus* models. Due to the incompleteness of the fossil material, the main body axis is based on a composite of *P.* cf. *kevani* CAMSM J. 35990 [23] and *P. funkei* PMO 214.135 [24], with the vertebral formula adopted from *P. brachyspondylus* CAMSM 35991 [25]. A comparison of vertebral dimensions in this study (with data from [23, 24]) supports the view that CAMSM J. 35990 and PMO 214.135 were of nearly equivalent size [24], assuming they shared similar body proportions along the main body axis. Consequently, the present reconstruction differentiates the overall body volume of the two specimens solely based on differences in limb size (Fig. S6).

3.1 Body length

As neither CAMSM J. 35990 and PMO 214.135 retains a complete vertebral sequence, the presacral vertebral formula from *P. brachyspondylus* CAMSM J. 35991 (19 cervical, 2 pectoral, and 24 dorsal vertebrae [25]) was adopted here. Among plesiosaurs, the position of the transition from cervical to dorsal vertebrae may exhibit intraspecific variation, whereas the total number of presacral vertebrae remains constant [26]. Given that *P. funkei* PMO 214.135 preserves three pectoral vertebrae [24], the presacral vertebral formula for the models were adjusted to 19 cervical, 3 pectoral, and 23 dorsal vertebrae.

The cervical series of *Pliosaurus* was previously divided into the "anterior cervicals" and the "posterior cervicals" due to their distinct morphology [27]. Given that CAMSM J. 35990 preserves a greater number of cervical vertebrae than PMO 214.135 [24], the neck length of the model was constructed based on their dimensions. The mean length of the cervical vertebrae was calculated using the following formula:

$$\text{mean length} = \frac{\text{mean length of anterior cervicals} \times 10 + \text{mean length of posterior cervicals} \times 9}{19} \quad (2)$$

The lengths of the three pectoral vertebrae in the models were set to 91, 100, and 100 mm, respectively. These values represent a composite derived from the measurable pectoral vertebrae preserved in both specimens [25, 24]. As the dorsal vertebral sequence of PMO 214.135 is more complete, the dorsal vertebral lengths for the models were calculated by comparing its dorsal vertebral measurements with those of *Brachauchenius lucasi* USNM 4989 [3] using Equation (1). The length of the first sacral vertebra was set equal to the average length of the dorsal vertebrae, while the intervertebral spacing of the presacral column was reconstructed according to the criteria outlined in the Extra Quantitative Criteria section above. The trunk length was estimated based on the restored $\frac{\text{pectorals+dorsals+sacral 1}}{\text{trunk}}$ proportion of *Sachicasaurus vitae*, and the tail length was calculated using the trunk-tail formula presented in the main text.

Since neither CAMSM J. 35990 nor PMO 214.135 preserves a complete skull [25, 24], the skull length (SKL) of the models was estimated using the skull-neck formula provided in the main text,

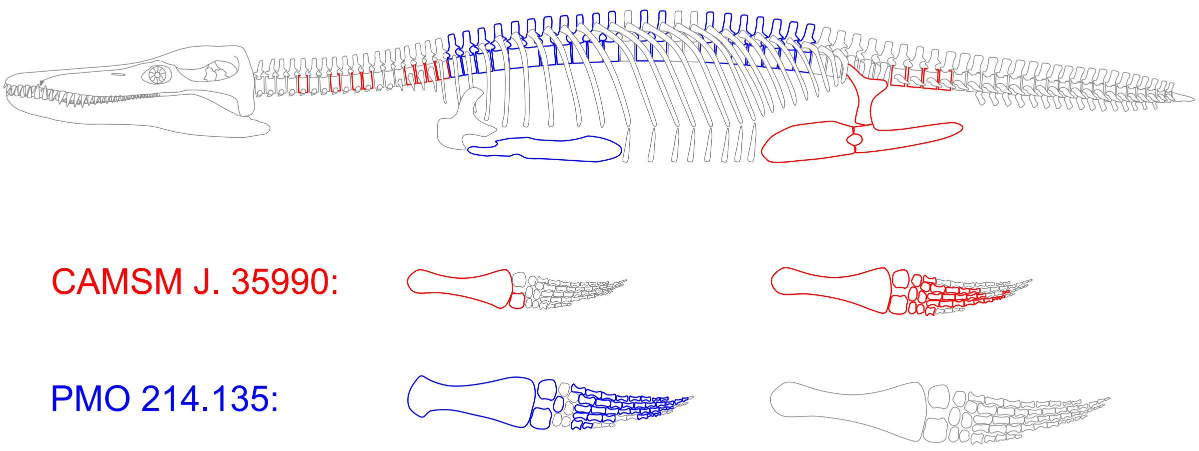

Figure 6: **The reconstruction for *Pliosaurus* cf. *kevani* and *Pliosaurus funkei*.** The highlighted lines indicate the fossil material used in reconstruction. Since the precise position of the preserved vertebrae within the spinal column can not be fully ascertained, the vertebral identification shown in the highlights is conjectural. Some fragmentary elements not utilized in this reconstruction, such as the cranial and rib fragments of PMO 214.135, have not been highlighted from the illustration.

yielding a result of 1850 mm. An alternative estimate, derived from the $\frac{\text{SKL}}{\text{cervical vertebral length}}$ ratio of *P. brachyspondylus* CAMSM J. 35991 [25], produced a value of 2168 mm. The average value (2009 mm) of these two results was taken and set as the SKL of the model. The basal condylar length (BCL) was then computed according to the $\frac{\text{BCL}}{\text{SKL}}$ proportion of *P. kevani* DORCM G. 13675 [28].

3.2 Ribcage and limbs

The pectoral and pelvic girdles of CAMSM J. 35990 were preserved but unfortunately damaged or lost after excavation [23]. Based on the original fossil illustration by [23], the width of its coracoid appears broadly comparable to that of the corresponding element in PMO 214.135 [29]. As no pelvic material is preserved in *P. funkei* [24], the dimensions provided in the 1959 line drawing of CAMSM J. 35990 [23] were adopted for the pelvic girdle of the models. This approach inevitably introduces a degree of uncertainty, though such compromises are unavoidable given the fragmentary nature of known material of *Pliosaurus*. Information regarding dorsal rib length is also nearly absent for both specimens [23, 24]. Therefore, the maximum rib arc length was calculated using the trunk-rib formula presented in the main text. The length of the ribs dorsal to the glenoid cavity was estimated by scaling the maximum rib arc length using the rib coefficient of *"Monquirasaurus" boyacensis* [13]. With these parameters acquired, the reconstruction workflow for the ribcage is identical to that applied to *Sachicasaurus vitae*.

As mentioned above, this study distinguishes the body volume of PMO 214.135 from that of CAMSM J. 35990 solely based on limb size. The propodial measurements for CAMSM J. 35990 were sourced from [23], and its total limb length was estimated using the previously established formula correlating distal propodial width with limb length [21]. Since no hindlimb material is preserved for PMO 214.135 [24], the dimensions of the femur were first approximated based on the humerus-to-femur ratio of CAMSM J. 35990, after which the same formula was applied to derive the

hindlimb length.

4 On some giant Jurassic pliosaurs

OUMNH PAL-J.010454, a mandible that was classified to *Stretosaurus* [23], *Liopleurodon* [30] and *Pliosaurus* [29] before, was restored as 2875 mm in length [23]. Length of the imperfect mandible before restoration was 7 feet (about 2134 mm), as briefly mentioned in [31]. The mandible is currently displayed in a glass exhibition case, precluding first-hand measurements, hence the photogrammetric method was used here to investigate its size. There exists a breakage behind the dentary on each ramus of the reconstructed mandible (pers. obs), and length of the mandible anterior to breakage matches the 7-feet measurement (measured from lateral view photos). It was argued in [23] that "...the posterior part of the left ramus has come to light... the total length would have been more than 3000 mm". Two associated mandibular fragments (OUMNH PAL-J.050376 and OUMNH PAL-J.050377) from the same individual were indeed recovered from the same pit as OUMNH PAL-J.010454 and match it in size [29]. It is not certain which specimen was referred to in [23], but if it was OUMNH PAL-J.050376, the anteroposterior length of the mandible should be around 2.6 m. Assuming an identical body proportion shared with *Pliosaurus* cf. *kevani* and a body density equivalent to surface sea water (1.027×10^3 kg/m^3), its body mass might reach 20 metric tons. It is not the only example indicating that Jurassic pliosaurs might reach 20 tons in mass. Some giant cervical vertebrae described in [32] and an isolated dorsal rib that is 122 cm in chord length [33] all indicate a similar body size.

5 Supplementary Table

Table 1: **Measurements and estimated values for the models used in this study.** Asterisked values denote estimated dimensions. Unless otherwise noted, trunk length and intervertebral spacing for neck length were reconstructed following the criteria outlined in the Extra Quantitative Criteria section, while tail and limb lengths were estimated using the regression equations mentioned in the main text. All measurements are in millimeters (mm). Abbreviations: SW, skull width; SH, skull height; SKL, skull length; HumL, humerus length; HumW, humerus distal width; ForeL, forelimb length; FemL, femur length; FemW, femur distal width; HindL, hindlimb length; CoraW, coracoid width; PubWA, pubic width at the acetabulum.

Taxon	element	size	element	size	note
Albertonectes vanderveldei TMP 2007.011.0001 [9]	SW	*183	HumW	226	SKL estimated using the skull-neck formula; skull porportions based on *Styxosaurus* SDSM 451 [34].
	SH	*171	ForeL	1464	
	SKL	*413	FemL	380	
	neck	7131	FemW	224	
	trunk	2205	HindL	*1267	
	tail	2384	CoraW	315	

	HumL	400	PubWA	360	
Aristonectes quiriquinensis SGO.PV.957 [35, 36]	SW	*498	HumW	270	SW and SKL based on the reconstruction from [36]. SH restored according to the skull reconstruction for *Morturneria seymourensis* [37]. PubWA based on the CoraW/PubWA ratio of *Thalassomedon haningtoni* DMNH 1588 [38]. FemL based on the HumL/HumW ratio of SGO.PV.957 [35]. Tail length based on the body proportion of SGO.PV.260 [36].
	SH	*323	ForeL	2288	
	SKL	*768	FemL	*531	
	neck	*3590	FemW	287	
	trunk	2773	HindL	*2271	
	tail	*3054	CoraW	423	
	HumL	500	PubWA	*436	
Hydrotherosaurus alexandrae UCMP 33912 [38]	SW	*170	HumW	270	SW and SH based on the skull reconstruction from [38].
	SH	*181	ForeL	*1529	
	SKL	351	FemL	360	
	neck	*4759	FemW	237	
	trunk	*1888	HindL	*1341	
	tail	*1811	CoraW	285	
	HumL	390	PubWA	323	
Styxosaurus SDSM 451 [34]	SW	186	HumW	265	
	SH	174	ForeL	1660	
	SKL	420	FemL	365	
	neck	*6270	FemW	245	
	trunk	*2396	HindL	1730	
	tail	2213	CoraW	310	
	HumL	380	PubWA	360	
Thalassomedon haningtoni DMNH 1588 [38, 39]	SW	252	HumW	290	Intercaudal cartilage set to 18% of the vertebral length according to *Aristonectes quiriquinensis* SGO.PV.260 [36].
	SH	258	ForeL	*1643	
	SKL	510	FemL	440	
	neck	*6566	FemW	257	
	trunk	*3215	HindL	1611	
	tail	*1680	CoraW	435	
	HumL	440	PubWA	448	
Vegasaurus molyi MLP 93-I-5-1 [40]	SW	*123	HumW	235	SKL estimated using the skull-neck formula. Skull proportions based on *Styxosaurus* SDSM 451 [34]
	SH	*115	ForeL	*1330	
	SKL	*277	FemL	280	
	neck	*3052	FemW	190	

	trunk	*1414	HindL	*1074	
	tail	*1419	CoraW	270	
	HumL	298	PubWA	278	
Abyssosaurus nataliae MChEIO PM/1 [41, 42]	SW	*200	HumW	190	Skull dimensions based on the reconstruction from [42]
	SH	*236	ForeL	*1074	
	SKL	*300	FemL	470	
	neck	*2812	FemW	230	
	trunk	*1853	HindL	*1302	
	tail	*1783	CoraW	210	
	HumL	400	PubWA	235	
Cryptoclidus eurymerus NHMUK PV R2860 [43, 26]	SW	*120	HumW	214	Skull proportions and neck length based on the reconstruction from [26]. The forelimbs and hindlimbs restored according to NHMUK PV R8575 [21] and NHMUK PV R3703 [43], respectively.
	SH	*156	ForeL	*817	
	SKL	*295	FemL	270	
	neck	*1180	FemW	160	
	trunk	*1133	HindL	*886	
	tail	*990	CoraW	204	
	HumL	285	PubWA	244	
Dolichorhynchops osborni FHSM VP404 [15]	SW	194	HumW	187	Skull deformed due to taphonomy. SH estimated from the skull proportion of another individual KUVP 1300 [11]. The propodials are reversed in the museum mount [44]. Intercaudal cartilage set to 10% of the vertebral length, according to *Mauriciosaurus fernandezi* [6].
	SH	*178	ForeL	838	
	SKL	513	FemL	336	
	neck	*603	FemW	168	
	trunk	1062	HindL	813	
	tail	*789	CoraW	257	
	HumL	330	PubWA	257	
Martinectes bonneri KUVP 40002 [45]	SW	*290	HumW	320	SKL estimate using the skull-neck formula. SH and SW restored according to the skull proportions of KUVP 40001 [45].
	SH	*297	ForeL	*1346	
	SKL	*880	FemL	515	
	neck	815	FemW	300	
	trunk	*1653	HindL	1440	
	tail	*1619	CoraW	335	
	HumL	526	PubWA	353	
Mauriciosaurus fernandezi	SW	152	HumW	105	SH based on the skull proportions of

INAH CPC RFG 2544 P.F.1. [6]	SH	*124	ForeL	510	*Dolichorhynchops osborni* KUVP 1300 [11].
	SKL	390	FemL	180	
	neck	442	FemW	85	
	trunk	588	HindL	465	
	tail	*679	CoraW	125	
	HumL	170	PubWA	134	
Polycotylus latipinnis YPM 1125 (data from the research notes of Samuel P. Welles, provided by Bruce Schumacher)	SW	*305	HumW	230	SKL estimated based on the skull-neck formula and the SKL/cervical vertebral lengths of SDSM 23020 [44]. SW restored according to the SW/SKL ratio of SDSM 23020 [44]. SH restored according to the skull of *Dolichorhynchops osborni* KUVP 1300 [11]. Femur dimensions restored according to the humerus/femur proportions of LACM 129639 [46]. ForeL and HindL lengths based on the HumL/ForeL and FemL/HindL ratios of *D. osborni* FHSM VP404 [15], as the results based on formula appearing too long.
	SH	*239	ForeL	*1016	
	SKL	*705	FemL	*428	
	neck	*1179	FemW	*215	
	trunk	*1620	HindL	*1036	
	tail	*1592	CoraW	270	
	HumL	400	PubWA	300	
Kronosaurus queenslandicus MCZ 1285 [20]	SW	*1107	HumW	*289	Basal condylar length (BCL; snout tip to occipital condyle) 2210 mm [3]. SW restored based on the SW/BCL ratio of the skull reconstruction from [3]. Mandibular length estimated from the BCL/mandible ratio of the same reconstruction. The quadrate region extensively modified, with the SKL estimated from the SKL/mandible of KK F0630 [47]. SH restored according to the skull of *Peloneustes philarchus* NHMUK PV R4058 [17]. Humerus dimensions restored according to the humerus/femur proportions of QM F10113 [3].
	SH	*909	ForeL	*1662	
	SKL	*2508	FemL	1060	
	neck	*1471	FemW	400	
	trunk	*3615	HindL	2300	
	tail	*3128	CoraW	590	
	HumL	*766	PubWA	610	
Liopleurodon ferox GPIT-RE-3184 [48]	SW	*443	HumW	218	Basal condylar length (BCL; snout tip to occipital condyle) 920 mm [48]. Other skull dimensions estimated based on the skull reconstruction from [49]. Neck length measured from the side view photo of the mount.
	SH	*412	ForeL	*1233	
	SKL	*1064	FemL	520	
	neck	*1090	FemW	285	
	trunk	*1948	HindL	*1614	
	tail	*1859	CoraW	330	
	HumL	402	PubWA	367	
"Monquirasaurus" boyacensis MJACM 1 [50]	SW	1060	HumW	321	Exact neck length unavailable since some anterior cervicals being obscured by the skull. The combined length of the rest cervicals and pectorals 908 mm. CoraW and PubWA estimated from the CoraW/ischium width and PubWA/ischium width ratio of *Kronosaurus queenslandicus* MCZ 1285, respectively [3].
	SH	*1004	ForeL	1870	
	SKL	2450	FemL	977	
	neck	/	FemW	401	
	trunk	3010	HindL	2208	
	tail	*2681	CoraW	*658	

	HumL	799	PubWA	*678	
Peloneustes philarchus GPIT-RE-3182 [48]	SW	*331	HumW	206	SW and SH restored according to the skull proportion of NHMUK PV R8574 and R4058, respectively [17].
	SH	*329	ForeL	*1165	
	SKL	790	FemL	425	
	neck	747	FemW	205	
	trunk	*1483	HindL	1210	
	tail	*1398	CoraW	320	
	HumL	380	PubWA	330	
Pliosaurus cf. *kevani* CAMSM J.35990 [23]	SW	*971	HumW	308	SKL based on the skull-neck formula and the skull/cervical vertebral lengths ratio of *Pliosaurus brachyspondylus* CAMSM J. 35991 [25]. Skull proportions based on the reconstruction for *Pliosaurus kevani* from [28].
	SH	*805	ForeL	*1745	
	SKL	*2009	FemL	960	
	neck	*1713	FemW	360	
	trunk	*3327	HindL	*2042	
	tail	*2917	CoraW	650	
	HumL	840	PubWA	686	
Pliosaurus funkei PMO 214.135 [24]	SW	*971	HumW	400	SKL based on the skull-neck formula and the skull/cervical vertebral lengths ratio of *P. brachyspondylus* CAMSM J. 35991 [25]. Skull proportions based on the reconstruction for *P. kevani* from [28]. Femur dimensions based on the humerus/femur proportions of CAMSM J. 35990 [23].
	SH	*805	ForeL	*2270	
	SKL	*2009	FemL	*1248	
	neck	*1713	FemW	*468	
	trunk	*3327	HindL	*2657	
	tail	*2917	CoraW	650	
	HumL	1000	PubWA	*686	
Sachicasaurus vitae MP111209-1 [2]	SW	1330	HumW	365	SH restored according to the skull of *Peloneustes philarchus* NHMUK PV R4058 [17]. CoraW estimated from the CoraW/PubWA ratio of *Kronosaurus queenslandicus* MCZ 1285 [20].
	SH	*1032	ForeL	*2019	
	SKL	2520	FemL	1000	
	neck	1156	FemW	470	
	trunk	3775	HindL	*2669	
	tail	*3244	CoraW	*616	
	HumL	870	PubWA	637	
Stenorhynchosaurus munozi VL17052004-1 [51]	SW	580	HumW	225	SH restored according to the skull proportion of *Peloneustes philarchus* NHMUK PV R4058 [17].
	SH	*441	ForeL	1480	
	SKL	1360	FemL	810	

	neck	1066	FemW	250	
	trunk	2077	HindL	1575	
	tail	*1962	CoraW	337	
	HumL	665	PubWA	372	
Macroplata tenuiceps NHMUK PV R5488 [52]	SW	220	HumW	152	
	SH	143	ForeL	*858	
	SKL	536	FemL	350	
	neck	1066	FemW	150	
	trunk	1168	HindL	*847	
	tail	1470	CoraW	238	
	HumL	320	PubWA	231	
Meyerasaurus victor SMNS 12478 [53]	SW	205	HumW	193	SH restored according to the proportion of the skull reconstruction for *Rhomaleosaurus* [1].
	SH	*159	ForeL	1110	
	SKL	370	FemL	385	
	neck	817	FemW	157	
	trunk	1100	HindL	1041	
	tail	1163	CoraW	200	
	HumL	420	PubWA	211	
Microcleidus tournemirensis MMM J. T. 86-100 [54]	SW	110	HumW	91	Skull dorsoventrally crushed [54]. SH restored according to the proportion of the skull reconstruction for *Microcleidus homalospondylus* [55].
	SH	*106	ForeL	*513	
	SKL	250	FemL	208	
	neck	2024	FemW	99	
	trunk	934	HindL	*555	
	tail	*1002	CoraW	129	
	HumL	208	PubWA	150	
Seeleyosaurus guilelmiimperatoris SMNS 12039 [7]	SW	*105	HumW	130	Inferred length from snout tip to retroarticular process 210 mm. Other skull dimensions restored according to the skull reconstruction from [56].
	SH	*93	ForeL	790	
	SKL	*190	FemL	256	
	neck	1342	FemW	116	
	trunk	940	HindL	886	
	tail	1182	CoraW	122	
	HumL	280	PubWA	141	
Brancasaurus	SW	89	HumW	130	Intercervical cartilage set to 7.2% of

brancai GPMM A3.B4 [57]	SH	106	ForeL	*734	the vertebral lengths, according to *Seeleyosaurus guilelmiimperatoris* SMNS 12039 due to their similarity in cervical count. Intercaudal cartilage set to 18% of the vertebral lengths, according to *Aristonectes quiriquinensis* SGO.PV.260 [36].
	SKL	252	FemL	215	
	neck	*1265	FemW	124	
	trunk	*840	HindL	770	
	tail	*729	CoraW	137	
	HumL	230	PubWA	140	
Nichollssaura borealis TMP 1994.122.0001 [58]	SW	133	HumW	104	PubWA inferred from the position of the acetabulum and the medial border of the left ischium. CoraW estimated according to the CoraW/PubWA ratio of *Brancasaurus brancai* GPMM A3.B4 [57].
	SH	106	ForeL	530	
	SKL	244	FemL	177	
	neck	640	FemW	109	
	trunk	875	HindL	532	
	tail	812	CoraW	*132	
	HumL	203	PubWA	135	
Plesiopterys wildi MH 7 [59]	SW	78	HumW	113	SH restored according to the skull reconstruction for *Seeleyosaurus guilelmiimperatoris* from [56].
	SH	*88	ForeL	686	
	SKL	188	FemL	213	
	neck	1081	FemW	113	
	trunk	842	HindL	717	
	tail	913	CoraW	118	
	HumL	214	PubWA	124	

References

[1] Adam S. Smith and Roger B. J. Benson. Osteology of *Rhomaleosaurus thorntoni* (Sauropterygia: Rhomaleosauridae) from the Lower Jurassic (Toarcian) of Northamptonshire, England. *Monographs of the Palaeontographical Society*, 168(642):1–40, oct 2014.

[2] María Eurídice Páramo-Fonseca, Cristian David Benavides-Cabra, and Ingry Esmirna Gutiérrez. A new large pliosaurid from the Barremian (Lower Cretaceous) of Sáchica, Boyacá, Colombia. *Earth Sciences Research Journal*, 22(4):223–238, 2018.

[3] Colin Richard McHenry. *Devourer of gods: the palaeoecology of the Cretaceous pliosaur Kronosaurus queenslandicus*. PhD thesis, University of Newcastle, 2009.

[4] Samuel Paul Welles. A new elasmosaur from the Eagle Ford Shale of Texas: systematic description. *Fondren Science Series*, 1, 1949.

[5] Pernille V Troelsen. *Mobility and hydrodynamic implications of the long neck in plesiosaurs*. PhD thesis, Liverpool John Moores University, 2018.

[6] Eberhard Frey, Eric W. A. Mulder, Wolfgang Stinnesbeck, Héctor E. Rivera-Sylva, José Manuel Padilla-Gutiérrez, and Arturo Homero González-González. A new polycotylid plesiosaur with extensive soft tissue preservation from the early Late Cretaceous of northeast Mexico. *Boletín de la Sociedad Geológica Mexicana*, 69(1):87–134, 2017.

[7] Sven Sachs, Daniel Madzia, Miguel Marx, Aubrey J. Roberts, Oliver Hampe, and Benjamin P. Kear. The osteology, taxonomy, and phylogenetic placement of *Seeleyosaurus guilelmiimperatoris* (Plesiosauroidea, Microcleididae) from the Lower Jurassic Posidonia Shale of Germany. *The Anatomical Record*, February 2025.

[8] Sven Sachs, Benjamin P. Kear, and Michael J. Everhart. Revised vertebral count in the "longest-necked vertebrate" *Elasmosaurus platyurus* Cope 1868, and clarification of the cervical-dorsal transition in Plesiosauria. *PLOS ONE*, 8(8):e70877, 2013.

[9] Tai Kubo, Mark T. Mitchell, and Donald M. Henderson. *Albertonectes vanderveldei*, a new elasmosaur (Reptilia, Sauropterygia) from the Upper Cretaceous of Alberta. *Journal of Vertebrate Paleontology*, 32(3):557–572, may 2012.

[10] Courtney D. Richards. Plesiosaur body shape and its impact on hydrodynamic properties, 2011.

[11] Samuel Wendell Williston. *North American plesiosaurs: Part I*, volume 2. Field Columbian Museum, 1907.

[12] Adam Stuart Smith. *Anatomy and systematics of the Rhomaleosauridae (Sauropterygia: Plesiosauria)*. PhD thesis, National University of Ireland, University College Dublin, 2007.

[13] Leslie F. Noè and Marcela Gómez-Pérez. Giant pliosaurids (Sauropterygia; Plesiosauria) from the Lower Cretaceous peri-Gondwanan seas of Colombia and Australia. *Cretaceous Research*, 132:105122, apr 2022.

[14] Donald M. Henderson. Lost, hidden, broken, cut-estimating and interpreting the shapes and masses of damaged assemblages of plesiosaur gastroliths. *PeerJ*, 12:e17925, August 2024.

[15] Orville Bonner. An osteological study of *Nyctosaurus* and *Trinacromerum* with a description of a new species of *Nyctosaurus*. Master's thesis, Fort Hays State University, 1964.

[16] Richard Owen. Monograph of the fossil reptilia of the Liassic formations. part first. Sauropterygia. *Monographs of the Palaeontographical Society*, 17(75):1–40, 1865.

[17] Hilary F. Ketchum and Roger B. J. Benson. The cranial anatomy and taxonomy of *Peloneustes philarchus* (Sauropterygia, Pliosauridae) from the Peterborough Member (Callovian, Middle Jurassic) of the United Kingdom. *Palaeontology*, 54(3):639–665, May 2011.

[18] Robert O. Clark, F. Robin O'Keefe, and Sara E. Slack. A new genus of small polycotylid plesiosaur from the Upper Cretaceous of the Western Interior Seaway and a clarification of the genus *Dolichorhynchops*. *Cretaceous Research*, 157:105812, December 2023.

[19] Charles William Andrews. *A descriptive catalogue of the marine reptiles of the Oxford Clay: based on the Leeds Collection in the British Museum (Natural History), London, Part II*. British Museum (Natural History), 1913.

[20] Alfred Sherwood Romer and Arnold D. Lewis. A mounted skeleton of the giant plesiosaur *Kronosaurus*. *Brevoria Museum of Comparative Zoology*, (112):1–15, 1959.

[21] Laura Austin Sydes. Identifying behavioural and functional groups using a geometric morphometric analysis of plesiosaur flipper morphology, 2022.

[22] Luke E. Muscutt, Gareth Dyke, Gabriel D. Weymouth, Darren Naish, Colin Palmer, and Bharathram Ganapathisubramani. The four-flipper swimming method of plesiosaurs enabled efficient and effective locomotion. *Proceedings of the Royal Society B: Biological Sciences*, 284(1861):20170951, August 2017.

[23] L. B. Tarlo. *Stretosaurus* gen. nov., a giant pliosaur from the Kimmeridge Clay. *Palaeontology*, 2(1):39–55, 1959.

[24] Espen M. Knutsen, Patric S. Druckenmiller, and Jørn H. Hurum. A new species of *Pliosaurus* (Sauropterygia: Plesiosauria) from the Middle Volgian of central Spitsbergen, Norway. *Norwegian Journal of Geology*, 92:235–258, 2012.

[25] L. B. Tarlo. *Pliosaurus brachyspondylus* (Owen) from the Kimmeridge clay. *Palaeontology*, 1(4):283–291, 1959.

[26] David Seymour Brown. The English Upper Jurassic Plesiosauroidea (Reptilia) and a review of the phylogeny and classification of the Plesiosauria. 1981.

[27] Lambert Beverly Tarlo. *A review of the Upper Jurassic pliosaurs*. British Museum (Natural History), 1960.

[28] Roger B. J. Benson, Mark Evans, Adam S. Smith, Judyth Sassoon, Scott Moore-Faye, Hilary F. Ketchum, and Richard Forrest. A giant pliosaurid skull from the Late Jurassic of England. *PLoS ONE*, 8(5):e65989, may 2013.

[29] Espen M. Knutsen. A taxonomic revision of the genus *Pliosaurus* (Owen, 1841a) Owen, 1841b. *Norwegian Journal of Geology*, 92:259–276, 2012.

[30] L. F. Noè, Don T. J. Smith, and D. I. Walton. A new species of Kimmeridgian pliosaur (Reptilia; Sauropterygia) and its bearing on the nomenclature of *Liopleurodon macromerus*. *Proceedings of the Geologists' Association*, 115(1):13–24, 2004.

[31] Joseph Prestwich. *Geology: chemical, physical, and stratigraphical*, volume 2. Clarendon Press, 1888.

[32] David M. Martill, Megan L. Jacobs, and Roy E. Smith. A truly gigantic pliosaur (Reptilia, Sauropterygia) from the Kimmeridge Clay Formation (Upper Jurassic, Kimmeridgian) of England. *Proceedings of the Geologists' Association*, 134(3):361–373, June 2023.

[33] Yves Lepage, Eric Buffetaut, and Gilles Lepage. Qu'est-ce que *Tapinosaurus*? Lennier, Rabeck et les Grands Sauroptérygiens du Kimméridgien supérieur de la région Havraise (Normandie, France). *Bulletin de la Société géologique de Normandie et des amis du Muséum du Havre (2004)*, 96(1):27–59, 2009.

[34] S. P. Welles and James D. Bump. *Alzadasaurus pembertoni*, a new elasmosaur from the Upper Cretaceous of South Dakota. *Journal of Paleontology*, pages 521–535, 1949.

[35] Rodrigo A. Otero, Sergio Soto-Acuña, Frank Robin O'Keefe, José P. O'Gorman, Wolfgang Stinnesbeck, Mario E. Suárez, David Rubilar-Rogers, Christian Salazar, and Luis Arturo Quinzio-Sinn. *Aristonectes quiriquinensis*, sp. nov., a new highly derived elasmosaurid from the upper Maastrichtian of central Chile. *Journal of Vertebrate Paleontology*, 34(1):100–125, jan 2014.

[36] Rodrigo A. Otero, Sergio Soto-Acuña, and Frank R. O'keefe. Osteology of *Aristonectes quiriquinensis* (Elasmosauridae, Aristonectinae) from the upper Maastrichtian of central Chile. *Journal of Vertebrate Paleontology*, 38(1):e1408638, jan 2018.

[37] F. Robin O'Keefe, Rodrigo A. Otero, Sergio Soto-Acuña, Jose P. O'gorman, Stephen J. Godfrey, and Sankar Chatterjee. Cranial anatomy of *Morturneria seymourensis* from Antarctica, and the evolution of filter feeding in plesiosaurs of the Austral Late Cretaceous. *Journal of Vertebrate Paleontology*, 37(4):e1347570, July 2017.

[38] Samuel Paul Welles. Elasmosaurid plesiosaurs with description of new material from California and Colorado. *Memoirs of the University of California*, 13:125–254, 1943.

[39] Kenneth Carpenter. Revision of North American elasmosaurs form the Cretaceous of the western interior. *Paludicola*, 2(2):148, 1999.

[40] José P. O'Gorman, Leonardo Salgado, Eduardo B. Olivero, and Sergio A. Marenssi. *Vegasaurus molyi*, gen. et sp. nov. (Plesiosauria, Elasmosauridae), from the Cape Lamb Member (lower maastrichtian) of the Snow Hill Island Formation, Vega Island, Antarctica, and remarks on Wedellian Elasmosauridae. *Journal of Vertebrate Paleontology*, 35(3), May 2015.

[41] A. Yu. Berezin. A new plesiosaur of the family Aristonectidae from the Early Cretaceous of the center of the Russian platform. *Paleontological Journal*, 45(6):648–660, November 2011.

[42] A. Y. Berezin. Morphofunctional features of the plesiosaur *Abyssosaurus nataliae* (Plesiosauridea: Plesiosauria) in connection with adaptation to a deep-sea lifestyle. *Scientific Works of the State Nature Reserve "Prisursky"*, (34):56–70, 2019.

[43] Charles William Andrews. *A descriptive catalogue of the marine reptiles of the Oxford clay: Based on the Leeds Collection in the British Museum (Natural History), London*, volume 1. order of the Trustees, 1910.

[44] Bruce A. Schumacher and James E. Martin. *Polycotylus latipinnis* Cope (Plesiosauria, Polycotylidae), a nearly complete skeleton from the Niobrara Formation (early Campanian) of southwestern South Dakota. *Journal of Vertebrate Paleontology*, 36(1):e1031341, 2016.

[45] Dawn A. Adams. *Trinacromerum bonneri*, new species, last and fastest pliosaur of the Western Interior Seaway. *Texas Journal of Science*, 49(3):179, 1997.

[46] F. R. O'Keefe and L. M. Chiappe. Viviparity and K-Selected life history in a Mesozoic marine plesiosaur (Reptilia, Sauropterygia). *Science*, 333(6044):870–873, August 2011.

[47] Timothy Holland. The mandible of *kronosaurus queenslandicus* Longman, 1924 (Pliosauridae, Brachaucheniinae), from the Lower Cretaceous of northwest Queensland, Australia. *Journal of Vertebrate Paleontology*, 38(5):e1511569, September 2018.

[48] Hermann Linder. Beiträge zur Kenntnis der Plesiosaurier-Gattungen *Peloneustes* und *Pliosaurus*. *Geologische und Palaeontologishe Abhandlungen*, 1913.

[49] Leslie Francis Noè. *A taxonomic and functional study of the Callovian (Middle Jurassic) Pliosauroidea (Reptilia, Sauropterygia)*. PhD thesis, University of Derby, 2001.

[50] Oliver Hampe. Ein großwüchsiger Pliosauride (Reptilia: Plesiosauria) aus-der Unterkreide (oberes Aptium) von Kolumbien. *Courier Forschungsinstitut Senckenberg*, 145:1–32, 1992.

[51] María Eurídice Páramo-Fonseca, Marcela Gómez-Pérez, Leslie F. Noè, and Fernando Etayo-Serna. *Stenorhynchosaurus munozi*, gen. et sp. nov. a new pliosaurid from the Upper Barremian (Lower Cretaceous) of Villa de Leiva, Colombia, South America. *Revista de la Academia Colombiana de Ciencias Exactas, Físicas y Naturales*, 40(154):84, March 2016.

[52] Hilary F. Ketchum and Adam S. Smith. The anatomy and taxonomy of *Macroplata tenuiceps* (Sauropterygia, Plesiosauria) from the Hettangian (Lower Jurassic) of Warwickshire, United Kingdom. *Journal of Vertebrate Paleontology*, 30(4):1069–1081, jul 2010.

[53] Adam S Smith and Peggy Vincent. A new genus of pliosaur (Reptilia: Sauropterygia) from the Lower Jurassic of Holzmaden, Germany. *Palaeontology*, 53(5):1049–1063, 2010.

[54] Nathalie Bardet, Pascal Godefroit, and Jacques Sciau. A new elasmosaurid plesiosaur from the Lower Jurassic of southern France. *Palaeontology*, 42(5):927–952, October 1999.

[55] David Brown, Peggy Vincent, and Nathalie Bardet. Osteological redescription of the skull of *Microcleidus homalospondylus* (Sauropterygia, Plesiosauria) from the Lower Jurassic of England. *Journal of Paleontology*, 87(4):537–549, July 2013.

[56] Franziska Großmann. The taxonomic and phylogenetic position of the Plesiosauroidea from the Lower Jurassic Posidonia shale of south-west Germany. *Palaeontology*, 50(3):545–564, may 2007.

[57] Sven Sachs, Jahn J. Hornung, and Benjamin P. Kear. Reappraisal of Europe's most complete Early Cretaceous plesiosaurian: *Brancasaurus brancai* Wegner, 1914 from the "Wealden facies" of Germany. *PeerJ*, 4:e2813, dec 2016.

[58] Patrick S. Druckenmiller and Anthony P. Russell. Skeletal anatomy of an exceptionally complete specimen of a new genus of plesiosaur from the Early Cretaceous (Early Albian) of northeastern Alberta, Canada. *Palaeontographica Abteilung A*, 283(1-3):1–33, March 2008.

[59] Miguel Marx, Sven Sachs, Benjamin P. Kear, Mats E. Eriksson, Klaus Nilkens, and Johan Lindgren. A new specimen of *Plesiopterys wildi* reveals the diversification of cryptoclidian precursors and possible endemism within European Early Jurassic plesiosaur assemblages. *PeerJ*, 13:e18960, March 2025.
